# Impact of Halogen Substituent Nature and Position on the Structural and Energetic Properties of Carbamazepine Cocrystals with Meta‐Halobenzoic Acids: A Two‐Pathway Synthesis Study

**DOI:** 10.1002/cplu.202500474

**Published:** 2025-09-18

**Authors:** Artur Mirocki, Mattia Lopresti

**Affiliations:** ^1^ Faculty of Chemistry University of Gdańsk W. Stwosza 63 Gdańsk 80–308 Poland; ^2^ Department of Science and Technological Innovation University of Eastern Piedmont Viale Teresa Michel 11 15121 Alessandria Italy

**Keywords:** carbamazepine, crystal engineering, Hirshfeld surface analysis, hydrogen bonds, mechanochemistry

## Abstract

Crystal engineering provides effective strategies to produce pharmaceutical cocrystals, aimed at enhancing the physicochemical properties of active pharmaceutical ingredients. Herein, the structural and energetic properties of carbamazepine cocrystals with meta‐chlorobenzoic, meta‐bromobenzoic, and meta‐iodobenzoic acids are examined in depth, with particular focus on the influence of halogen substitution. A comparative assessment of solution‐based crystallization and mechanochemical synthesis via liquid‐assisted grinding provides insight into the viability of different synthetic methodologies. The crystallographic analysis reveals isostructurality among the three cocrystals, with lattice stability being modulated by the increasing atomic radius of the halogen substituent. Complementary techniques, including thermogravimetry, differential scanning calorimetry, Fourier transform infrared spectroscopy, and Hirshfeld surface analysis, further elucidate the intermolecular forces driving the formation of these crystalline phases. The lattice energy calculations offer a quantitative perspective on the role of halogen substitution in stabilization, enriching the understanding of fundamental crystal engineering principles relevant to pharmaceutical development.

## Graphical abstract

This study explores how halogen substitution in benzoic acid coformers affects the structure and stability of carbamazepine cocrystals. Isostructurality and enhanced lattice stability with heavier halogens are revealed, highlighting key supramolecular interactions. Mechanochemical synthesis proves efficient and eco‐friendly, offering insights for pharmaceutical crystal design.

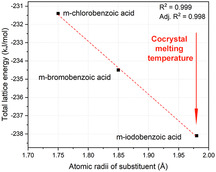

## 1 Introduction

The design and development of pharmaceutical cocrystals has emerged as a key strategy for modulating the physicochemical properties of active pharmaceutical ingredients (APIs), such as solubility, bioavailability, and stability.^[^ ^1^, ^2^, ^3^, ^4^, ^5^, ^6^, ^7^, ^8^, ^9^ ^–^ ^10^ ^]^ As an extension of crystal engineering principles, cocrystal formation is governed by the rational exploitation of supramolecular synthons:^[^ ^11^, ^12^, ^13^, ^14^, ^15^ ^–^ ^16^ ^]^ highly directional and predictable noncovalent interactions, such as hydrogen bonding, halogen bonding, and *π*‐stacking.^[^ ^17^, ^18^, ^19^, ^20^, ^21^, ^22^, ^23^ ^–^ ^24^ ^]^ The rational design of cocrystals enables the stabilization of API solid forms through interactions with coformers, leading to improved pharmaceutical performance.^[^ ^2^ ^]^

Carbamazepine (IUPAC name: 5H‐dibenzo[b, f]azepine‐5‐carboxamide), a widely used anticonvulsant for the treatment of epilepsy, trigeminal neuralgia, and bipolar disorder,^[^ ^25^, ^26^, ^27^ ^–^ ^28^ ^]^ is known for its multiple polymorphic and hydrated forms, which complicate its formulation and manufacturing processes.^[^ ^29^, ^30^ ^–^ ^31^ ^]^ Due to its polymorphic behavior, carbamazepine has been extensively studied as a model system in crystal engineering, particularly for cocrystal formation as a strategy to overcome these challenges.^[^ ^32^ ^,^ ^33^ ^]^ The self‐assembly of carbamazepine cocrystals is primarily dictated by supramolecular synthons, with amide‐to‐acid heterosynthons playing a dominant role in crystal packing.^[^ ^24^ ^,^ ^34^, ^35^ ^–^ ^36^ ^]^

The crystallization of carbamazepine cocrystals follows a stepwise mechanism: initial nucleation is driven by synthon formation, followed by molecular recognition and lattice stabilization.^[^ ^37^ ^]^ This process is influenced by factors such as solvent environment, temperature, and competing polymorphic forms, necessitating a comparative analysis of synthetic pathways to optimize cocrystal yield and purity.^[^ ^38^ ^]^

This study investigates how substituents of different sizes and positions on the aromatic ring influence the formation of crystalline structures, playing a role in the molecular recognition and lattice stabilization phases described above. Carbamazepine was reacted with counterparts capable of reliably forming synthons in solution (halobenzoic acids) and their structural isomers. It was observed that, under the same crystallization conditions, only the cocrystals of meta‐halobenzoic acids formed crystalline structures, all of which exhibited isostructurality.^[^ ^39^ ^]^

A structural, energetic, and physicochemical characterization of three novel cocrystals of carbamazepine with meta‐halobenzoic acids is presented. Such cocrystals were synthesized using two distinct approaches: solution‐based crystallization and mechanochemical synthesis via liquid‐assisted grinding (LAG). The mechanochemical method is an environmentally friendly method of obtaining crystalline materials. This synthesis significantly reduces the amount of liquid waste, such as organic solvents, and energy consumed, shortens synthesis time, enables scaling process, and contributes to increasing the bioavailability of drugs. Presence of small amounts solvent during LAG favors obtaining crystalline products and increases the reaction efficiency.^[^ ^40^, ^41^, ^42^, ^43^ ^–^ ^44^ ^]^

The isostructural nature of these three compounds, coupled with their synthesis through different methods, enabled a comprehensive multitechnique characterization, including single‐crystal X‐ray diffraction (SCXRD), powder X‐ray diffraction (XRPD), thermogravimetry (TG), differential scanning calorimetry (DSC), microscopy, mass spectrometry, elemental analysis, and infrared spectroscopy. Furthermore, their structural similarity enabled us to explore the energetic aspects of crystal stabilization through Hirshfeld surface analysis, fingerprint plots, and lattice energy calculations. Notably, for the first time, the energetic contribution of a substituent from the same group of the periodic table with increasing atomic radius to crystal lattice stabilization has been quantitatively assessed, shedding light on its potential role in crystallization processes.

## 2 Results and Discussion

### 2.1 Background and Rationale

**Figure 1:**
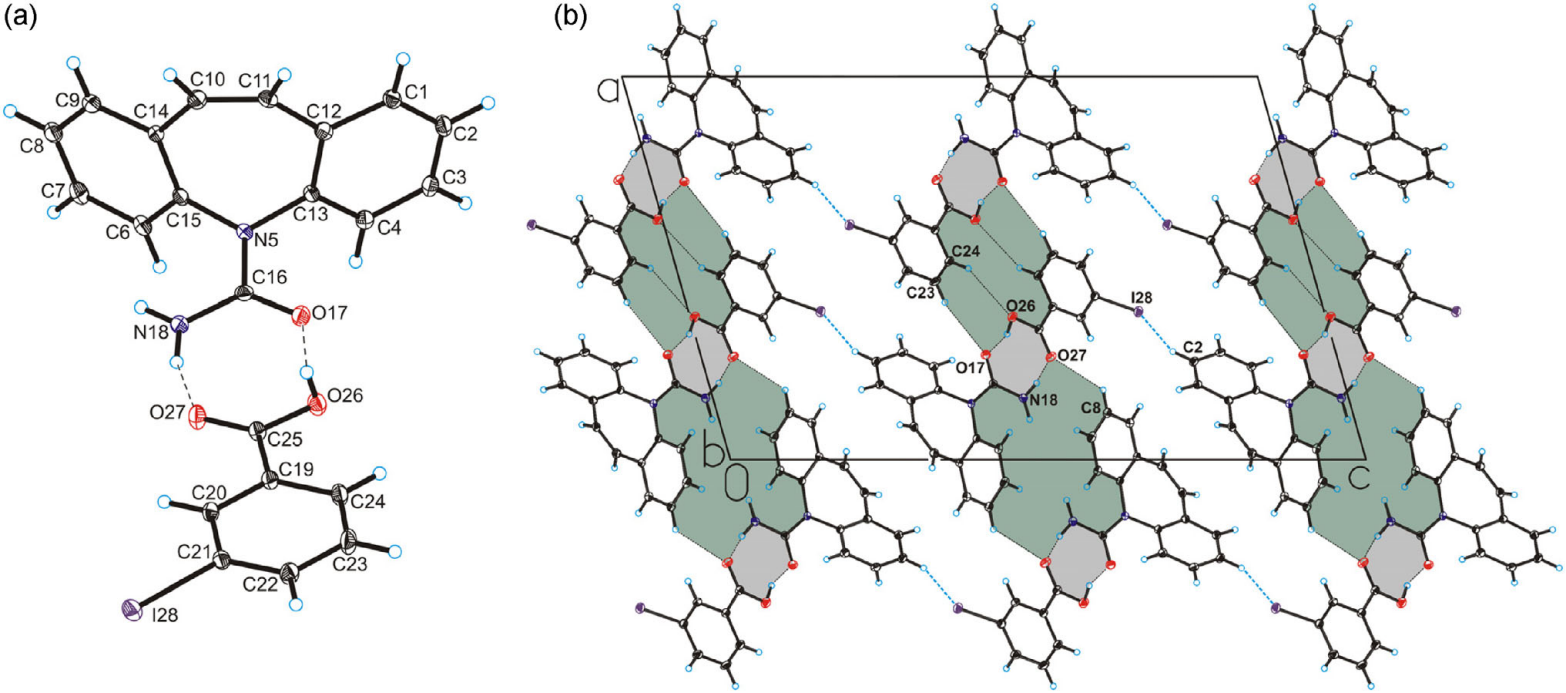
a) The asymmetric unit of compound **3*α*** showing the atom‐labeling scheme, b) crystal packing of compound 3*α* viewed along the b‐axis. The hydrogen bonds are represented by dashed black lines, the I—H interaction are represented by dashed blue lines (all three compounds are isostructural therefore only for m‐iodobenzoic acid cocrystal is reported, as the chlorine and bromine derivatives have the same the molecular structure and 3D framework structure, and they are reported in Figure S10 and S11, Supporting Information).

A review of the Cambridge Structural Database (CSD database, Version 2025.1.0) shows that 181 structures containing carbamazepine are deposited. Of these, there are 36 structures of carbamazepine and benzoic acids, and only one structure containing a halogen atom (REFCODE: OFEGIT).^[^ ^45^ ^]^ In order to fill the gap, and to have more insight on the formation of carbamazepine cocrystals, attempts were made to obtain carbamazepine crystals with ortho‐, meta‐, and para‐ halobenzoic acids using the slow solvent evaporation and the LAG methods (all pairs are reported in Table S1, Supporting Information) with the use various solvents or mixtures. The attempts made have shown that using both approaches it is possible to obtain compounds containing carbamazepine and meta‐halobenzoic acids in the form of cocrystals: carbamazepine and 3‐chlorobenzoic acid (1), carbamazepine and 3‐bromobenzoic acid (2), and carbamazepine and 3‐iodobenzoic acid (3) (**Figure** **1**). Attempts to obtain compounds containing carbamazepine and 3‐fluorobenzoic acid (carried out from various solvents or mixtures), using both methods were unsuccessful, because each time both substances crystallized separately. When ortho‐ and para‐halobenzoic acids were used, an amorphous material or a mixture of reagents was obtained, so under these conditions of synthesis, only compounds containing carbamazepine and meta‐halobenzoic acids were obtained. The similar situation was described by T. Oshikawa et al. in his studies on brucine.^[^ ^46^ ^]^ Similarly to the title compounds, the formation of cocrystals does not depend on the acidity of the halobenzoic acids. The inability to obtain crystalline materials from ortho‐ and para‐ substituted isomers is likely due to steric hindrance, both intra‐ and intermolecular, associated with the position of the substituents, which prevents efficient crystal packing. In contrast, meta‐substituted isomers can form tighter packing arrangements, thereby facilitating cocrystal formation.^[^ ^46^ ^]^

The intriguing observation that only the meta‐structural isomer yielded a crystalline structure, which was previously observed^[^ ^23^ ^]^ and cannot be easily explained, serves as a starting point for future research aimed at gaining a deeper understanding of the energetic insights that favor only certain species. This could contribute to the advancement of knowledge in the field of crystal engineering. In the following sections, the formation of three particularly stable isostructural species is utilized to investigate the interactions that define the chemical environment of the synthon. This investigation employs a combination of physicochemical characterization techniques, Hirshfeld surface analysis, and computational methods for lattice energy calculation.

### 2.2 Structure Solution and Packing Description

SCXRD measurements show that the crystals of **1*α***‐**3*α*** crystallize in the monoclinic *P*2_1_/c space group with one unit of carbamazepine molecule and one unit of 3‐halobenzoic acid molecule in the asymmetric unit and are isostructural (Figure 1, Table S2, Supporting Information). The lengths of C—O bonds range from 1.20 to 1.34 Å, indicating a proton transfer not occurring between the carboxylic group of halobenzoic acids molecules and carbamazepine molecule, which is also visible comparing to other compounds containing carbamazepine and benzoic acids (e.g., REFCODE: OFEGIT,^[^ ^45^ ^]^ MOXWEC,^[^ ^47^ ^]^ MOXVAX,^[^ ^47^ ^]^ FAYXOV^[^ ^48^ ^]^), where the lengths of C—O bonds range from 1.22 to 1.32 Å. The Kitaigorodskii type of packing index equal to 67,5% for compound **1*α*** and 68% for compounds **2*α*** and **3*α***. The carbamazepine molecule interacts with the halobenzoic acids molecules through the N_(amino)_—H…O_(carboxyl)_ and O_(carboxyl)_—H…O_(carbamazepine)_ hydrogen bonds forming a heterodimer (Figure 1, Table S3, Supporting Information). The same heterodimers are present in the structures of cocrystals of carbamazepine with other benzoic acids, such as benzoic acid (REFCODE: MOXVAX),^[^ ^47^ ^]^ 5‐chloro‐2‐hydroxybenzoic acid (REFCODE: OFEGIT),^[^ ^45^ ^]^ 2‐hydroxy‐4‐nitrobenzoic acid (REFCODE: FAYXOV),^[^ ^48^ ^]^ 3,5‐dinitrobenzoic acid (REFCODE: HORWIX)^[^ ^49^ ^]^ and other.^[^ ^34^ ^,^ ^50^, ^51^, ^52^ ^–^ ^53^ ^]^

**Figure 2:**
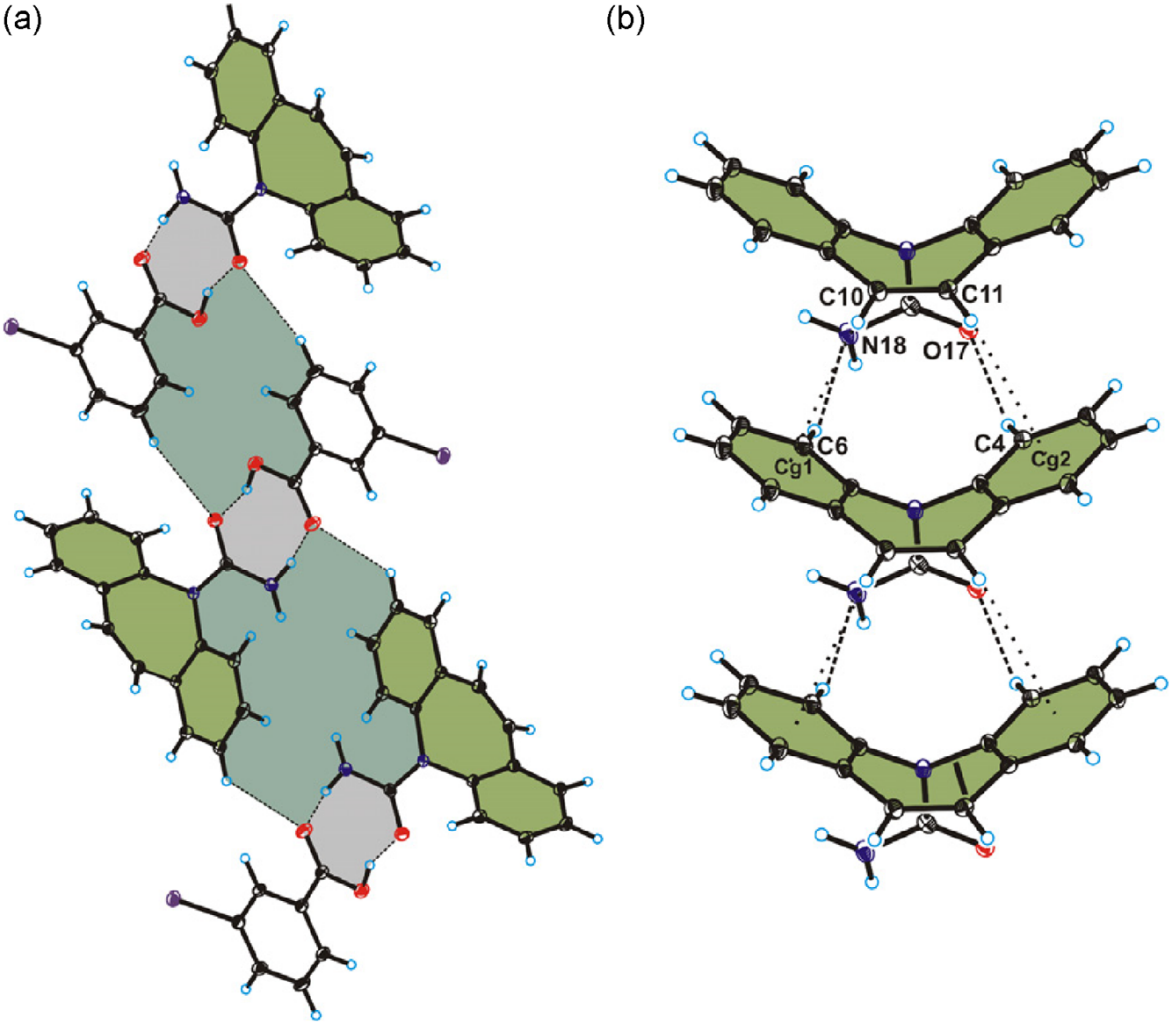
a) Crystal packing of compound 3*α* viewed along the b‐axis, b) the translation stack conformation of carbamazepine in compound 3*α* viewed along [111] direction. Hydrogen bonds are represented by dashed lines and the C–H···*π* interactions are represented by dotted lines.

In the crystals of compounds **1*α***‐**3*α***, the molecules of carbamazepine adopt the translation stack motif.^[^ ^47^ ^]^ The key interactions in this conformation are the C—H···N and C—H···O hydrogen bonds and C—H···*π* interactions (all three compounds are isostructural; therefore, only for m‐iodobenzoic acid cocrystal is reported in **Figure** **2**) (Figure S12 and Table S4‐S5, Supporting Information). Carbamazepine molecules are arranged parallel along the b‐axis (Figure 2a), forming stacks, as shown in Figure 2b.

The lengths of C—H···O hydrogen bonds (from 2.659 to 2.671 Å) between carbamazepine in translation stack motif are shorter than the lengths of C—H···N hydrogen bonds (from 2.742 to 2.749 Å) and the weak C—H···*π* interactions range from 3.072 to 3.123 Å in compound **1*α***‐**3*α***. Compared to the structures of compound **1*α***‐**3*α*** with structure of carbamazepine (REFCODE: CBMZPN11),^[^ ^54^ ^]^ in which the translation stack motif is present, the lengths of the C—H···O hydrogen bonds in compound **1*α***‐**3*α*** are longer than in carbamazepine (from 2.443 to 2.578 Å), but the lengths of the C—H···N hydrogen bonds and the weak C—H···*π* interactions are similar to carbamazepine (from 2.579 to 2.762 Å for hydrogen bonds and from 2.896 to 3.227 Å for C—H···*π* interactions) (Table S4, Supporting Information).

Analysis of the CSD Database^[^ ^55^ ^]^ shows that the inversion cup motif or translation stack motif involving carbamazepine molecules predominates in the structures of benzoic acids monosubstituted in the ortho or para position. The space group does not affect the type of motif occurrence (**Table** **1**).

**Table 1:** REFCODEs and types of packing motif in the structures of monosubstituted benzoic acids and carbamazepine.^[^ ^47^ ^,^ ^72^, ^73^, ^74^, ^75^, ^76^ ^–^ ^77^ ^]^

REFCODE	Types of packing motif	Space group	Position of the substituent in benzoic acid
INUZAU	Translation stack	*C*2/c	para
MOXVIF	Inversion cup	*P*2_1_/c	para
MOXVIF01	Inversion cup	*P*2_1_/n	para
MOXVIF03	Inversion cup	*P*2_1_	para
MOXWAY	Translation stack	*P*2_1_/n	ortho
RUTGOE	Inversion cup	*P*‐1	ortho
TAZRAO	Inversion cup	*P*‐1	ortho
TAZRAO01	Inversion cup	*P*‐1	ortho
XAQRAJ	Inversion cup	*C*2/c	para
XOXHEY	Translation stack	*P*2_1_/n	para
This work			
Compound 1	Translation stack	*P*2_1_/c	meta
Compound 2	translation stack	*P*2_1_/c	meta
Compound 3	translation stack	*P*2_1_/c	meta

Interestingly, comparing the compounds containing monosubstituted benzoic acids (REFCODE: INUZAU, MOXWAY, and XOXHEY), which carbamazepine occurs in the translation stack motif, the lengths of the C—H···O hydrogen bonds (from 2.461 to 2.608 Å) are shorter than in compound 1*α*‐3*α*, but the lengths of the C—H···N hydrogen bonds (from 2.676 to 2.782 Å) and the weak C—H···*π* interactions (from 2.973 to 3.142 Å) are similar to compound 1*α*‐3*α*. In the crystal structures of compounds 1*α*‐3*α*, adjacent heterodimers are connected by weak the C—H…O hydrogen bonds, forming a layer (Figure 1b). The adjacent layers are connected by the weak C—H···Cl, or C—H···Br, or C—H···I hydrogen bonds in compounds 1*α*‐3*α*, respectively, which additionally stabilize the crystal structures and form a 3D framework structure (Figure 1b), (the crystal packing of compound 1*α*‐3*α* is reported in Figure S11, Supporting Information).

### 2.3 XRPD Qualitative Analysis and Refinement of LAG Samples

Samples prepared *via* LAG underwent to characterization by XRPD analysis. The investigated samples included the original reagents treated with the LAG technique, and reagent pairs reported in Table S1, Supporting Information. For sake of brevity, only the successful crystallization attempts are detailed (namely, 1*β*, 2*β*, and 3*β*).

**Figure 3:**
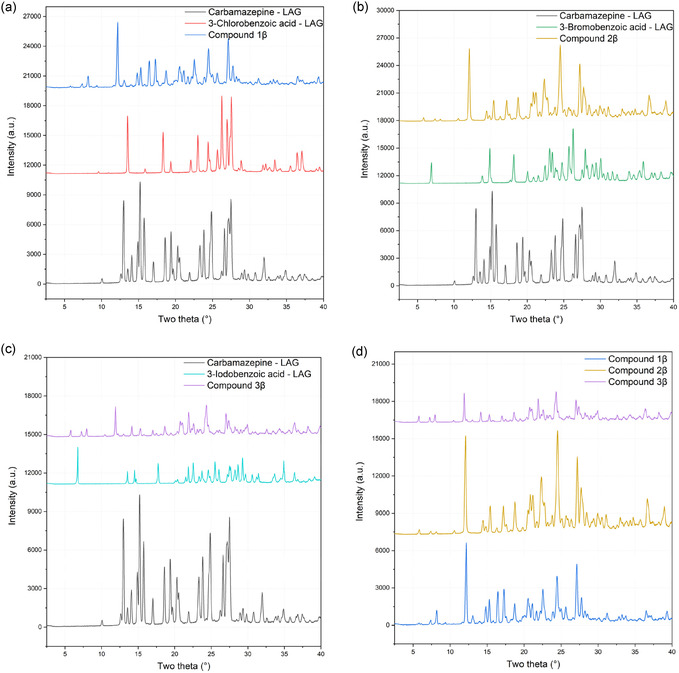
Qualitative comparison between compounds **1*β***, **2*β***, and **3*β*** with the respective reagents a–c) and between each other d).

In **Figure** **3**, the diffraction patterns of reagents after LAG treatment in comparison with their respective products are reported. Qualitative analysis of the peaks positions reveals the transformation induced by the mechanochemical process, resulting in the formation of three compounds, different from the patterns of the initial reagents (Figure 3a–c). Figure 3d further compares the diffraction patterns of the three products, highlighting their remarkable similarity and suggesting that samples obtained via LAG coincide with those solved by SCXRD.

Rietveld refinement confirms the structural consistency between products obtained via LAG and those solved by SCXRD, underlining the stability of the cocrystal and the efficacy of employing alternative methodologies for product synthesis. Structural refinements (Figure S13, Supporting Information) were carried out by considering background, zero‐point error, scale factor, the Lorentzian component of the crystallite size, and the Lorentzian component of microstrain, the latter being particularly relevant when refining structures obtained via LAG. Cell parameters were refined only after these variables had been optimized, resulting in minor differences in unit cell dimensions, within ±0.02 Å per edge. For compound 1*β* (Figure 3a), an excess of carbamazepine is present in the mechanochemically derived product, along with an unidentified impurity possibly stemming from secondary reaction. Conversely, compound 2*β* (Figure 3b) shows no impurities, highlighting the potential for achieving a 100% yield through mechanochemical synthesis. Finally, in compound 3*β* (Figure 3c), an excess of carbamazepine, estimated at 11%, is noted. Additionally, a potential phase not attributable to the reagents is observed, as indicated by a small peak around 12° in 2 theta. Notably, in all LAG products, improved refinement results can be achieved including preferred orientations compensation along *h* = 1, *k* = 0, l = −1.

### 2.4 Thermal Behavior

**Figure 4:**
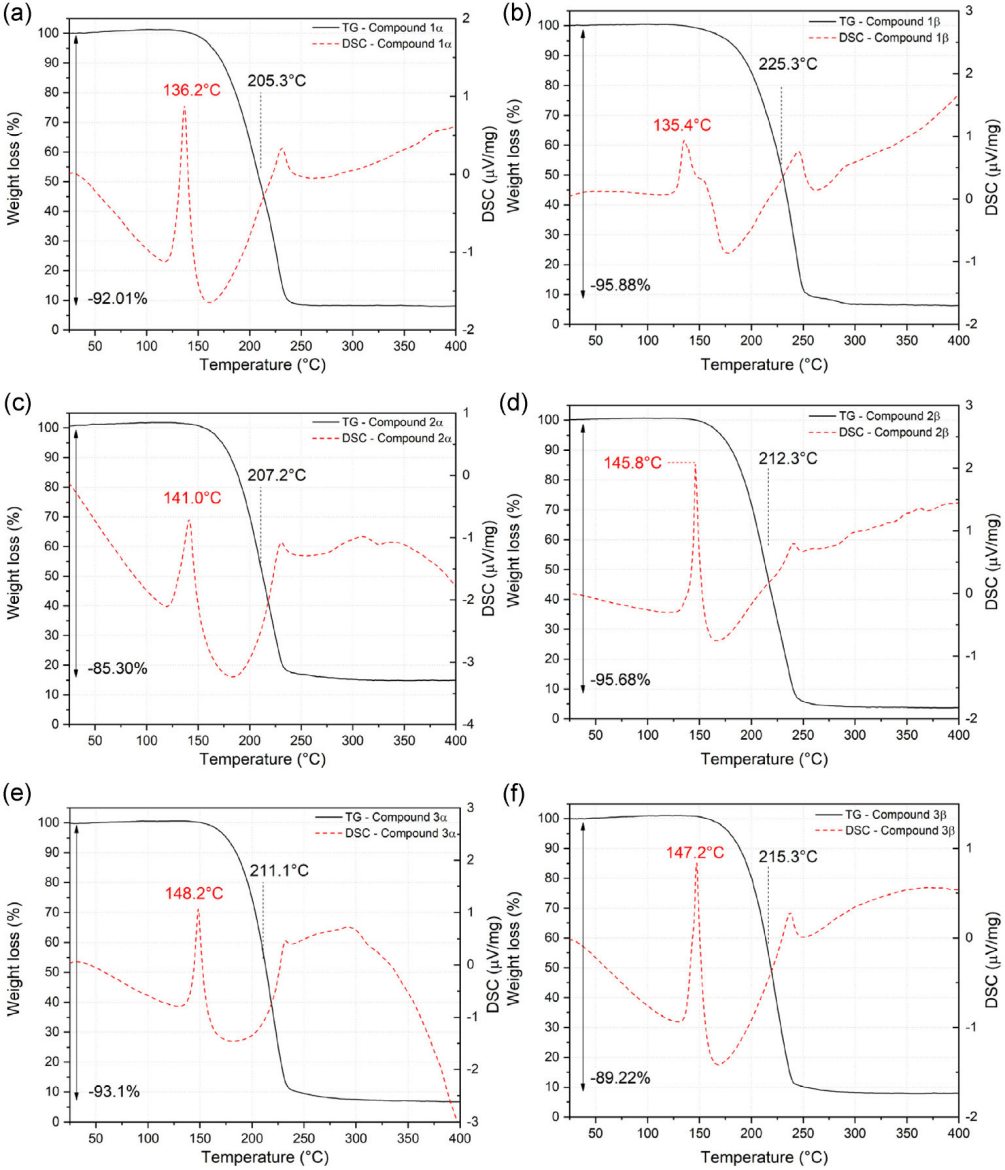
TG and DSC results for each compound. On the left, from top to bottom: a) **1*α***, c) **2*α***, and e) **3*α***; on the right, from top to bottom: b) **1*β***, d) **2*β***, and f) **3*β***. Black curve: TG profile; red curve: DSC profile.

Samples **1**‐**3**, from both the ***α*** and ***β*** sets, were characterized by thermal analysis using TG and DSC to determine the melting point and the thermal degradation profile. **Figure** **4** shows the thermograms for each sample: on the left column, the samples from the *α* set, and on the right, the corresponding samples from the *β* set.

In all the thermograms (Figure 4) the same behavior can be observed for all compounds. The distinctive shape of the DSC curve, featuring a peak around 140 °C followed by a "U"‐shaped profile, indicates that the sample underwent thermal decomposition immediately after melting. The TG profiles for all samples confirm this interpretation, as only a single, unimodal weight loss is observed within this temperature range, consolidating the interpretation of the DSC curve. In the case of compund **2**, the temperature difference between the ***α*** and ***β*** samples exceeds 4 °C. However, this can be justified by uncertainties in temperature measurement, differences in crystallinity, and the distinct thermal history of the samples due to the LAG procedure. The species previously suggested as impurities or secondary‐phase products can be excluded based on the DSC thermograms, which display no additional peaks that would indicate the presence of extra phases. Figure 4 shows a phase transition temperature around 136 °C for **1**, 143 °C for **2,** and 148 °C for **3**.

### 2.5 Attenuated Total Reflectance–Fourier Transform Infrared Spectroscopy (ATR‐FTIR) Characterization

**Figure 5:**
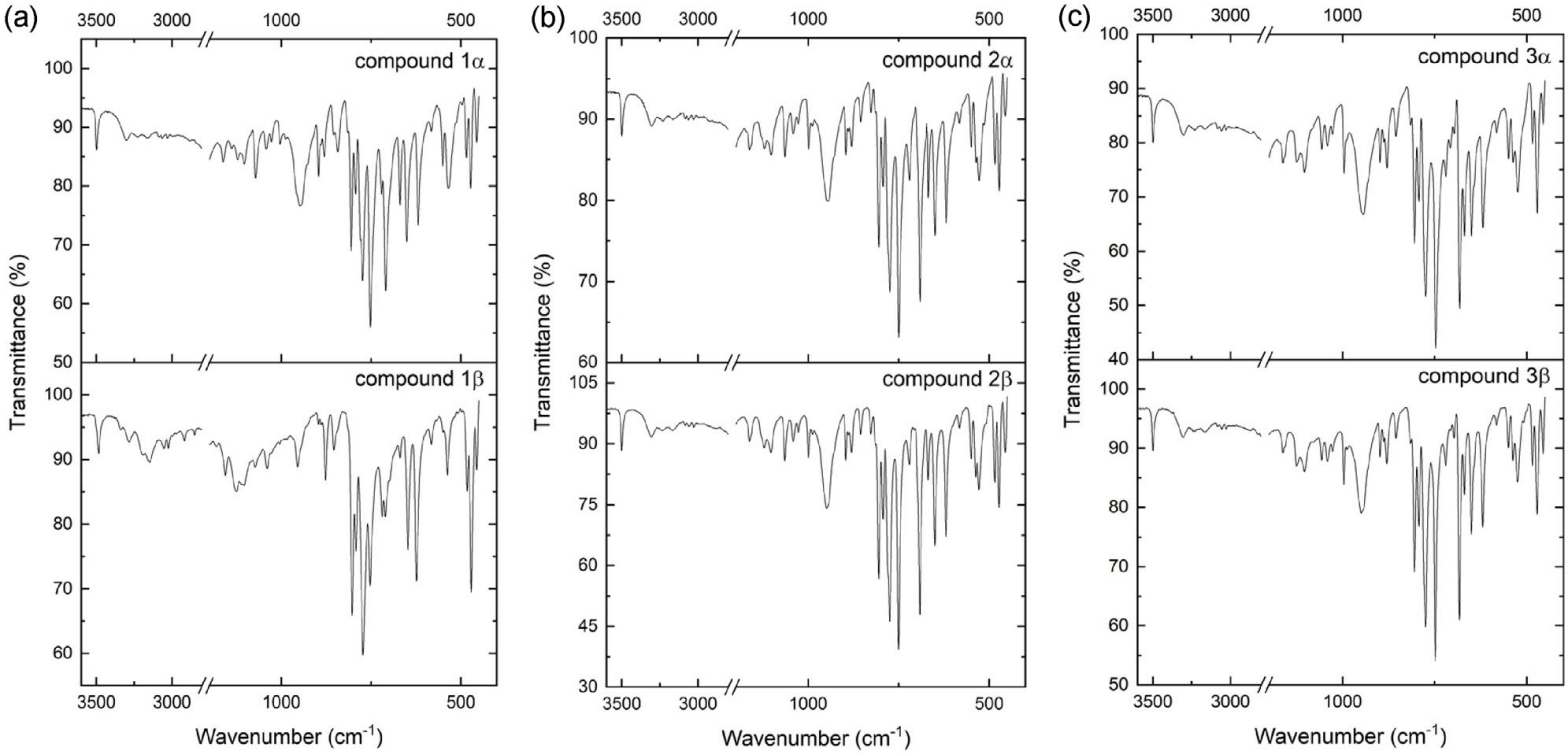
The ATR‐FTIR spectra in the range of 3500–400 cm^−1^ of a) carbamazepine and 3‐chlorobenzoic acid; b) carbamazepine and 3‐bromobenzoic acid; and c) carbamazepine and 3‐iodobenzoic acid.

In **Figure** **5**, all ATR‐FTIR spectra collected on products are reported. Comparing the ATR spectra of single crystals and powders of the title compounds, it is visible that the results obtained are identical, which also confirms that both pathways for obtaining compounds **1**, **2,** and **3** lead to the same products (the detailed description of the spectra can be found Figure S14, Supporting Information)

### 2.6 Hirshfeld Surface and Fingerprint Plot Analysis

Hirshfeld surface analyses were performed exploiting CrystalExplorer 21 on each solved structure after final refinement with high‐resolution settings. This analysis generates as an output a 3D surface that allows the quick and intuitive visualization of the molecular surroundings of the packing in the crystal structure. An in‐depth description of the surface calculation is reported in the ESI file. Because the newly identified cocrystals are isostructural, a detailed comparison between them was possible, enabling a quantitative assessment of the impact of various halogen substituents on the chemical environment of the meta‐substituted acid. Such an approach has been previously proposed by McKinnon and colleagues.^[^ ^56^ ^]^

In each structure examined, carbamazepine exhibited consistent percentage values for all potential interactions, and for this reason, it was only listed once in **Table** **2**. Conversely, it was noted that the interactions reported for halogenic acids varied from one another.

**Table 2:** Percentage of the interactions among all the possible pairs of atoms in the structures. Carbamazepine values are constant for each structure; therefore, it is reported only once.

Molecule	C···C	C···H	C···N	C···O	C···X	H···C	H···H	H···N	H···O	H···X	N···C	N···H	N···N	N···O	N···X
m‐chlorobenzoic acid	3.6%	8.8%	0.0%	3.8%	2.6%	6.1%	30.0%	0.0%	8.3%	2.0%	–	–	–	–	–
m‐bromobenzoic acid	3.7%	8.5%	0.0%	3.6%	2.8%	5.8%	29.4%	0.0%	7.9%	2.1%	–	–	–	–	–
m‐iodobenzoic acid	3.6%	8.3%	0.0%	3.3%	2.8%	5.9%	28.1%	0.0%	7.5%	2.2%	–	–	–	–	–
carbamazepine	1.3%	19.5%	0.0%	0.4%	0.0%	14.5%	44.3%	0.7%	6.3%	4.7%	0.0%	0.7%	0.0%	0.5%	0.0%
Molecule	O···C	O···H	O···N	O···O	O···X	X···C	X···H	X···N	X···O	X···X	C ···all	H ···all	N ···all	O ···all	X ···all
m‐chlorobenzoic acid	2.4%	11.7%	0.8%	0.4%	0.2%	2.6%	15.7%	0.0%	0.3%	0.7%	18.8%	46.4%	–	15.5%	19.3%
m‐bromobenzoic acid	2.4%	11.5%	0.7%	0.4%	0.1%	3.2%	16.2%	0.0%	0.3%	1.4%	18.6%	45.2%	–	15.1%	21.1%
m‐iodobenzoic acid	2.2%	11.3%	0.7%	0.3%	0.1%	3.6%	17.7%	0.0%	0.3%	2.1%	18.0%	43.7%	–	14.6%	23.7%
carbamazepine	0.6%	6.3%	0.0%	0.2%	0.0%	–	–	–	–	–	21.2%	70.5%	1.2%	7.1%	–

The data presented in Table 2 illustrates that intermolecular interactions primarily stem from the hydrogens within the two molecules found in the asymmetric unit. For carbamazepine, these account for over 70% of the total interactions. Concerning m‐substituted acids, it is noteworthy that hydrogens consistently constitute a significant portion of the total contacts (m‐chlorobenzoic acid: 46.4%; m‐bromobenzoic acid: 45.2%; m‐iodobenzoic acid: 43.7%).

**Figure 6:**
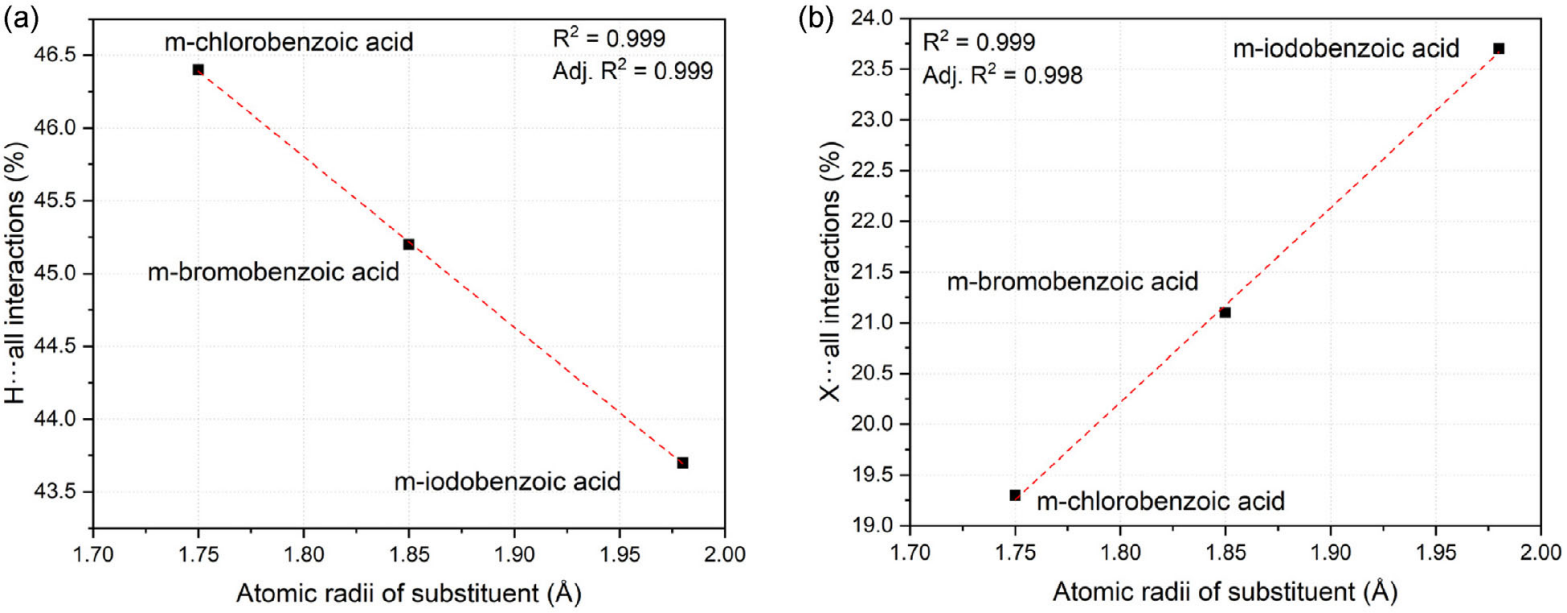
Atomic radius of the halogen substituent dependence on m‐halogenic acid percentual contacts in cocrystals. a) Atomic radius of substituent versus internal hydrogens with every external atom contact, b) atomic radius of substituent versus internal halogen with every external atom contacts.

A decrease, proportional to the atomic radius *r* of the m‐substituted halogen on benzoic acid (*r*_*X*_), can be observed for the percentage of H···all calculated intermolecular interactions (**Figure** **6**a). Conversely, there's proportional increase between *r*_*X*_ and the percentage of X···all interactions (Figure 6b).

The primary contributions to the decreasing trend in hydrogen‐based interactions can be distinguished between H···H and H···O interactions. Conversely, interactions showing a proportional increase can be distinguished between X···H and X···X interactions. Overall, the aggregated data trend would suggest that heavier substituents on benzoic acid result in more expanded structures with larger distances between molecules in the asymmetric unit.

#### 2.6.1 Carbamazepine

**Figure 7:**
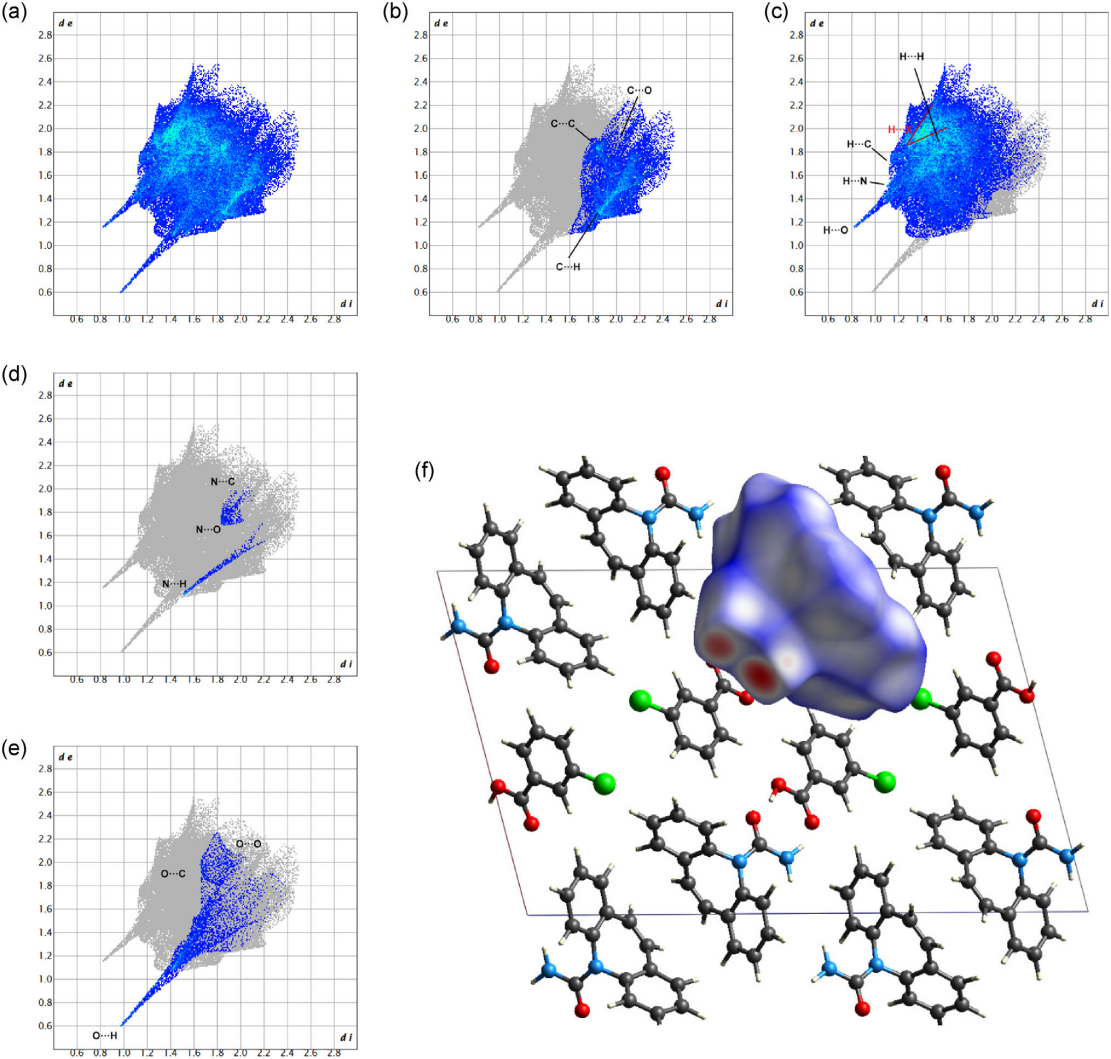
Hirshfeld surface and fingerprint plots of carbamazepine: a) complete fingerprint plots; b) filtered for C contacts; c) filtered for H contacts; d) filtered for N contacts; e) filtered for O contacts; and f) Hirshfeld surface for carbamazepine along b‐axis.

In **Figure** **7**, the Hirshfeld surface and element‐filtered fingerprint plots of carbamazepine are presented. Notably, the most prominent short contacts identified are N—H···O and O···H—O, indicative of interactions occurring at the on‐the‐plane interface between the amidic group of carbamazepine and the carboxylic group of the acid. This confirms that the strongest interaction inside the structure is given by the formation of the heterosynthon with the benzoic acid counterpart. Additionally, the presence of a homosynthon involving two carbamazepine molecules is noteworthy. This synthon, characterized by N—H···O hydrogen bonds between adjacent carbamazepine molecules, is frequently observed in various carbamazepine cocrystals and polymorphs, contributing to their structural stability, as reported in the previous sections. The competition between the carbamazepine···carbamazepine homosynthon and the carbamazepine···acid heterosynthon plays a crucial role in dictating the final crystalline arrangement, with the latter being favored due to the strong carboxyl‐amide interactions. Interestingly, due to the specific packing arrangement of the molecules, a carbamazepine/m‐halobenzoic acid system exhibits equivalent configurations situated directly above and below the referenced arrangement. Consequently, other notable short interactions are observed, such as H6···N’ and H4···O’, further contributing to the understanding of the molecular interactions and particular stability within this system.

#### 2.6.2 Meta‐Halobenzoic Acids

**Figure 8:**
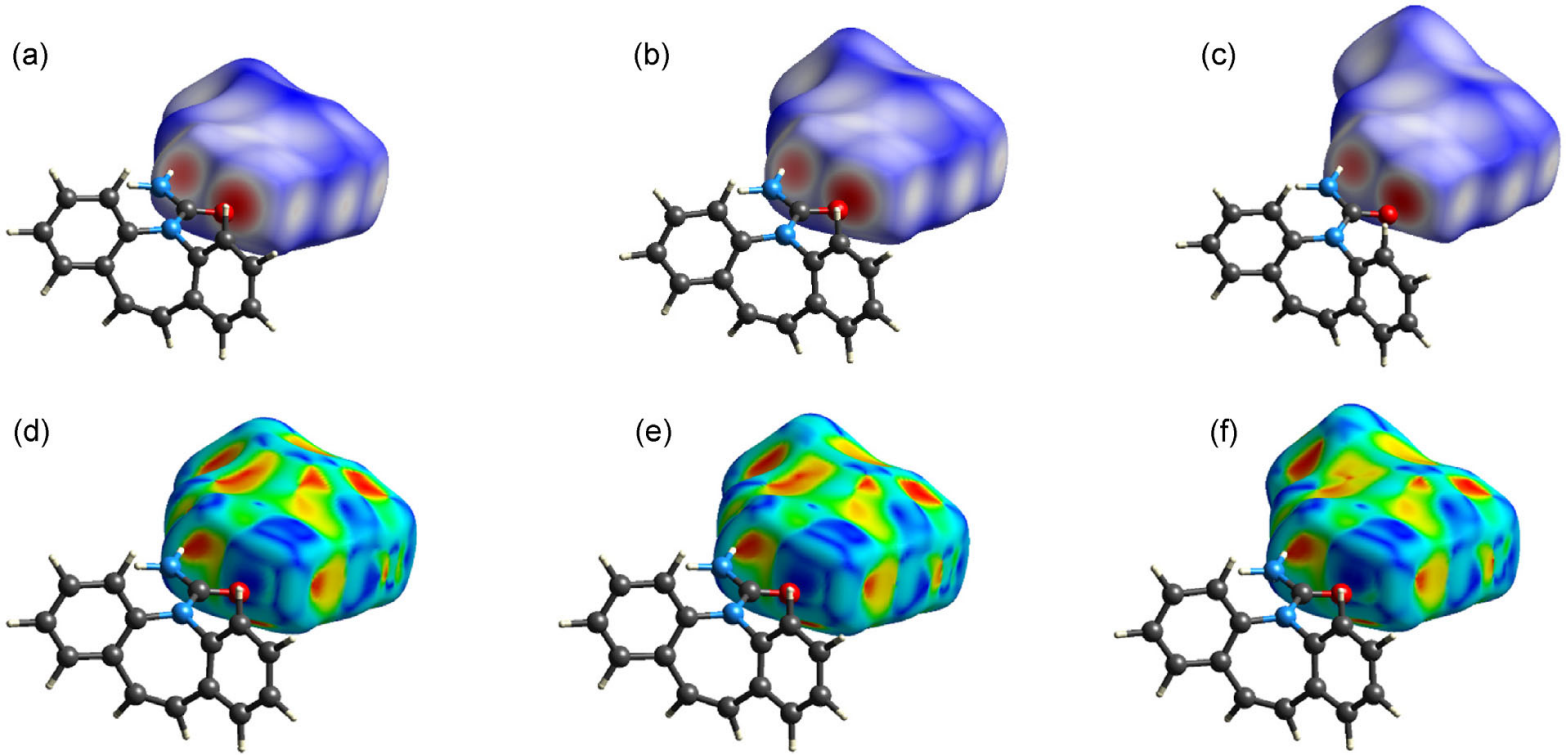
Calculated 3D Hirshfeld surfaces for the three m‐halobenzoic acids: a,d) 3D Hirshfeld surface of m‐chlorobenzoic acid, b,e) 3D Hirshfeld surface of m‐bromobenzoic acid, and c,f) 3D Hirshfeld surface of m‐iodobenzoic acid. Top row: d‐norm surface; bottom row: shape index surface.

The 3D Hirshfeld surfaces of the three acids appear visually like each other, but with color tones transitioning from deep blue to white proportionally with the *r*_*X*_ as defined in the previous sections. This can be easily observed in **Figure** **8**. In the bottom row of Figure 8, the relative shape index surfaces are also reported for a better visualization of the hue changes in the surface of the three halobenzoic acids.

**Figure 9:**
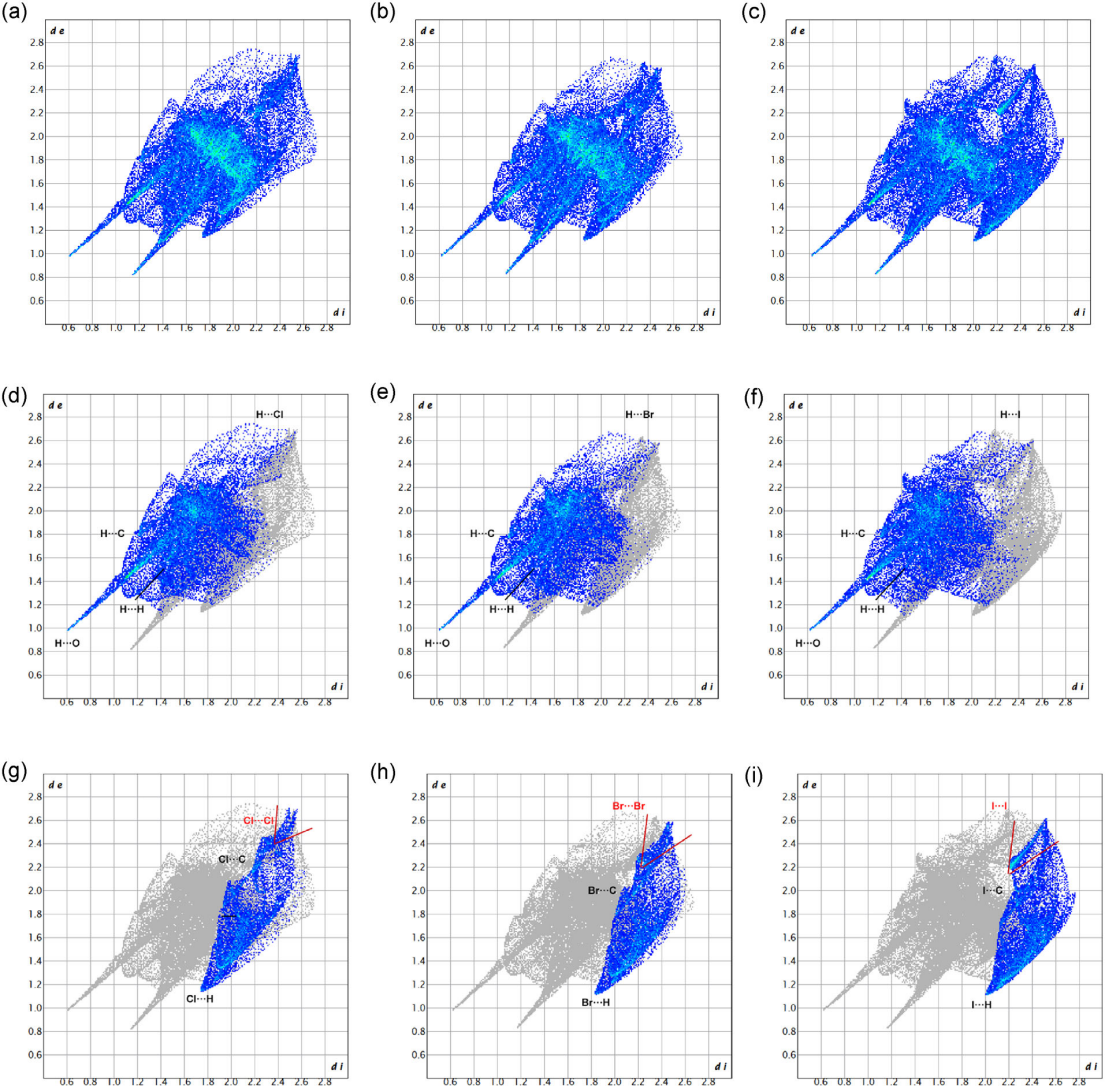
Complete fingerprint plot for each m‐halobenzoic acid of compound **1–3** in a–c), respectively; filtered fingerprint plot for hydrogen interactions of compound **1–3** in d–f), respectively, the filtered for the halogen interactions of compound **1–3** in g–i), respectively.

In **Figure** **9**, fingerprint plots are reported for each halobenzoic acid. The diagrams are divided into columns: m‐chlorobenzoic acid on the left, m‐bromobenzoic acid in the center, and m‐iodobenzoic acid on the right. In the graphs of the top row (Figure 9a–c), complete fingerprint plots are shown for each molecule. The main short contacts between the carboxylic and amidic groups remain consistent across all structures. However, the contacts attributed to the substituent halogen vary in terms of *d*_*i*_ and *d*_*e*_ values (chlorine: *d*_*i*_ = 1.7/ *d*_*e*_ = 1.1; bromine:*d*_*i*_ = 1.8/ *d*_*e*_ = 1.1; iodine: *d*_*i*_ = 2.0/ *d*_*e*_ = 1.1). The central row of Figure 9 shows a decrease in hydrogen‐based contacts in the molecular environment with an increase in *r*_X,_ while on the bottom row of Figure 9, a noticeable shift towards lowers *d*_*i*_ and *d*_*e*_ values is observed for the spike related to halogen–halogen contacts, along with an overall increase in contacts.

#### 2.6.3 Energy Frameworks and Total Lattice Energy

Energy models, derived from fitting dispersion‐corrected density functional theory (DFT) energies using B3LYP level of theory and DGDZVP basis set, help in understanding crystal packing. Total lattice energies for each solved structure were calculated as indicated in the work of Thomas et al.^[^ ^57^ ^]^ and are reported in **Table** **3**.

**Table 3:** Total energy for each molecule in the asymmetric unit and total lattice energy.

Substituent	r_*X*_ [Å]	m‐Halobenzoic acid [kJ mol^‐1^]	Carbamazepine [kJ mol^−1^]	Total lattice energy [kJ mol^−1^]
Chlorine	1.75	−88.9	−142.5	−231.4
Bromine	1.85	−92.3	−142.3	−234.5
Iodine	1.98	−96.9	−141.2	−238.1

**Figure 10:**
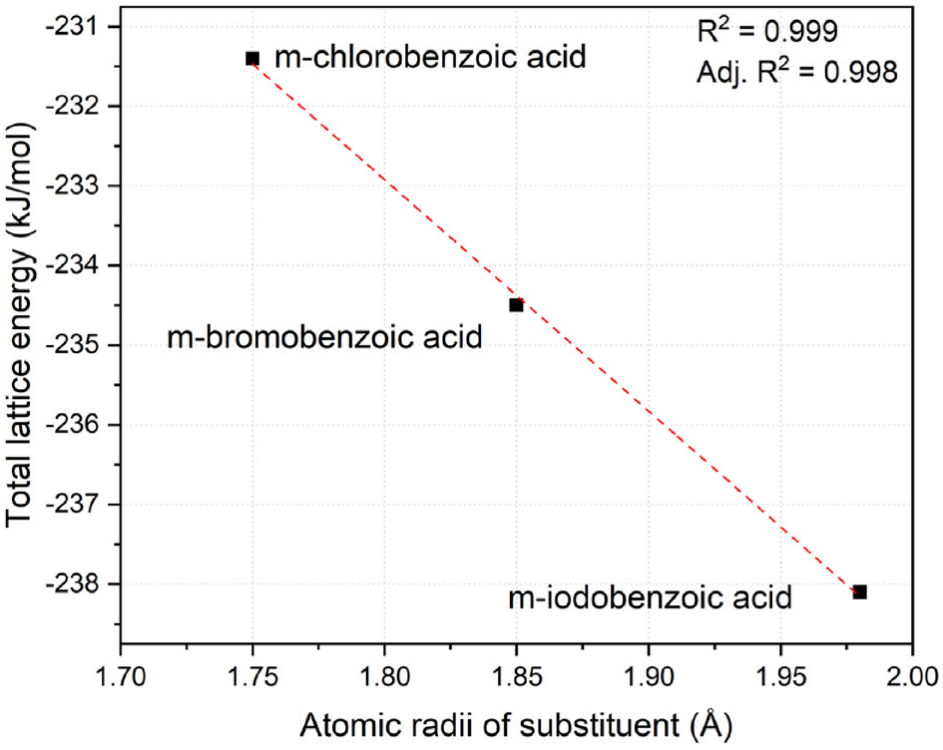
Atomic radii of the halogen substituents versus the total lattice energy computed using crystal explorer.

It is observed that the total energies calculated for carbamazepine are almost constant, which is consistent with what has been observed by analyzing the fingerprint plots and 3D Hirshfeld surfaces. In contrast, decreasing energies are observed with increasing atomic radius of the substituting halogen (*r*_*X*_) on benzoic acid, with a minimum value in the case of iodine. This suggests that large halogens exert a stabilizing effect within the crystal lattice, which is also confirmed by the rise in the percentage of halogen contacts with the neighboring molecules observed in Table 2. **Figure** **10** confirms the existence of a linear trend between the total lattice energy and *r*_*X*_, further confirming the aforementioned observations. Moreover, the trend in melting points of the compounds observed in Figure 4 is a further confirmation of the enhanced stability of the crystal structure imparted by heavier substituent atoms. While the increased lattice stability observed with larger halogens has been primarily attributed to size‐related steric effects and enhanced dispersion interactions, consistent with the role of heavier atoms as symmetry‐defining elements in crystal structures, the potential contribution of electronic effects, such as lone pair availability and ring deactivation must be acknowledged. Although the present dataset does not allow for a quantitative deconvolution of these factors, the observed trends likely arise from a synergistic interplay between steric and electronic contributions.

**Figure 11:**
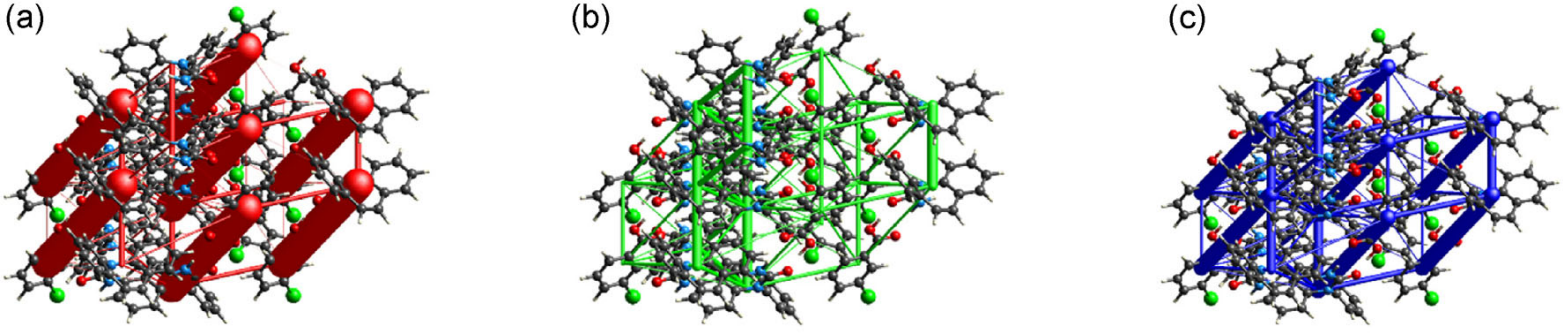
Energy frameworks for the carbamazepine/m‐chlorobenzoic acid (**1**) solved structure. a) Coulomb energy; b) dispersion energy; and c) total energy. Only the for m‐chlorobenzoic acid cocrystal is reported, as the bromine and iodine derivatives have the same energy frameworks.

Energy frameworks, visualized as cylinders representing interaction energies, aid in analyzing supramolecular architecture. These frameworks reveal anisotropy in molecular packing, crucial for understanding mechanical behavior like bending and shearing in crystals. Overall, energy frameworks provide a powerful tool to comprehend the complex interplay of forces influencing molecular crystal structures in various directions. In **Figure** **11**, the energy frameworks calculated for **1** are depicted. The energy frameworks of the other two isostructural co‐crystals are not shown as they appear to be perfectly identical in appearance. It can be observed that the contributions due to Coulomb interactions are positioned in the interface plane between the carboxylic and amide groups, confirming a strong electrostatic interaction. Of lesser magnitude, but still significant, are the Coulomb interactions between carbamazepine molecules along the translation axis. Along the same direction, cylinders representing the highest lattice dispersion energies are observed. The two combinations of the two parameters, reported in Figure 11, illustrate how the total energy is predominantly oriented in the aforementioned plane and that there are areas where interactions are weaker.

## 3 Conclusion

The role of halogen substitution of benzoic acid coformers is shown to influence both the formation and stability of carbamazepine cocrystals. The selective crystallization observed exclusively for the meta‐substituted isomers underscores the significance of molecular recognition and supramolecular interactions in directing the assembly of crystalline architecture beyond the role of the synthon formation. The isostructurality among the three cocrystals highlights a recurring structural motif, while the progressive increase in lattice stability with the atomic radius of the halogen substituent suggests a stabilizing effect associated with larger substituents. Mechanochemical synthesis via liquid‐assisted grinding emerges as a viable and sustainable alternative to solution‐based crystallization, offering advantages in terms of efficiency and environmental impact. The substitution of benzoic acid in the meta position may determine not only the arrangement of carbamazepine molecules relative to each other and, consequently, the packing of crystal structures, but also affect the length of bonds between carbamazepine molecules. This knowledge provides insight into the nature of the crystal structure of carbamazepine and benzoic acid compounds, contributing to a deeper understanding of the principles governing crystal engineering and pharmaceutical material design. Future studies aimed at investigating the physical or functional properties of the title compounds, such as solubility, stability, or bioavailability, are envisioned to fully assess their pharmaceutical potential.

## 4 Experimental Section

4.1

4.1.1

##### Synthesis and crystallization from solution and LAG of compound 1‐3

The compounds obtained by solvents evaporation were designated as **1*α***, **2*α***, and **3*α***, while the compounds obtained by the LAG method were designated as **1*β***, **2*β***, and **3*β***. All the chemical compounds were purchased from Sigma–Aldrich.

The carbamazepine (0.040 g, 0.169 mmol) and 3‐chlorobenzoic acid (0.026 g, 0.169 mmol) or 3‐bromobenzoic acid (0.034 g, 0.169 mmol) or 3‐iodobenzoic acid (0.042 g, 0.169 mmol) were dissolved in 12 cm^3^ of a dichloromethane/ethanol/water mixture (5:5:2 v/v) and boiled for 30 min. The solutions were allowed to evaporate for seven days to give white crystals of **1*α***, **2*α***, and **3*α***, respectively (Figure S1‐S3, Supporting Information). The results of elemental analysis and matrix‐assisted laser desorption/ionization time‐of‐flight (MALDI‐TOF) mass spectra are in ESI. (Figure S4‐S6, Supporting Information). Quantities and pairs for all the unsuccessful crystallization attempts are reported in Table S1, Supporting Information.

The carbamazepine (0.040 g, 0.169 mmol) and 3‐chlorobenzoic acid (0.026 g, 0.169 mmol) or 3‐bromobenzoic acid (0.034 g, 0.169 mmol) or 3‐iodobenzoic acid (0.042 g, 0.169 mmol) were gently ground together in an agate mortar with 20 drops (about 1 ml) of dichloromethane/ethanol/water mixture (5:5:2 v/v), then treated in an oven at 100 °C for 3 h for complete solvent evaporation, resulting in compounds **1*β***, **2*β***, and **3*β***. The results of elemental analysis and MALDI‐TOF mass spectra are in ESI. (Figure S7‐S9, Supporting Information).

##### SCXRD

The SCXRD data were collected on an Oxford Diffraction Gemini R ULTRA Ruby CCD diffractometer with CuK*α* (*λ *= 1.5418 Å) radiation at *T* = 295(2) K. The instrument was operated at 40 mA of current and 40 kV of electric potential. The lattice parameters were obtained by least‐squares fit to the optimized setting angles of the reflections collected by means of CrysAlis CCD.^[^ ^58^ ^]^ Data were reduced using CrysAlis RED software^[^ ^58^ ^]^ and applying multiscan absorption correction. The three investigated structures were solved with direct methods that carried out refinements by full‐matrix least‐squares on *F* ^2^ using the SHELXL‐2017/1 program.^[^ ^59^ ^]^ H atoms belonging to O/N atoms were located on a difference Fourier map and refined freely with U_iso_(H) = 1.5/1.2U_eq_(O/N). H atoms bound to C atoms were placed according to the principles of the crystal chemistry and refined using a riding model with *d*(C—H) = 0.93–0.98 Å and U_iso_(H) = 1.2U_eq_(C). The structures were validated using the PLATON program.^[^ ^60^ ^]^ The atomic radii were taken from PLATON program.^[^ ^60^ ^]^ Structure visualization and molecular graphics were produced using the following software: ORTEP II,^[^ ^61^ ^]^ PLUTO‐78,^[^ ^62^ ^]^ and Mercury.^[^ ^63^ ^]^ Full crystallographic details of the structures reported in this paper have been deposited with the Cambridge Crystallographic Data Centre (deposition no. CCDC 2,429,571, CCDC 2,429,572, and CCDC 2,429,573 for **1*α***, **2*α***, and **3*α***, respectively). As the crystals of compounds **1*α***‐**3*α*** (especially **1*α***) were of nonoptimal quality (Figure S1‐S3, Supporting Information) and exhibited very weak X‐ray diffraction, repeated measurements were performed on different crystals; in Table S2, Supporting Information the dataset with the best R_int_ is reported. In contrast, the crystals of compounds **1*β* ‐3*β*** were not suited for SCXRD, as the product was obtained by grinding the two reactants. For this reason, the latter compounds have been characterized by PXRD (Figures S15‐S17, supporting information).

##### XRPD

The XRPD data were collected with a Bruker D8 Advance powder diffractometer equipped with a LynxEye XE‐T strip detector and CuK*α* (*λ *= 1.5406 Å) radiation source. The goniometer radius was set to 280 mm (default distance). The X‐ray tube was set at operating conditions of 40 mA current and 40 kV electric potential. On the primary track, automated divergent slits and primary Soller slits of 2.5° opening were positioned. Automated slits were set to obtain a constant irradiated area of the sample with a width of 12 mm. On the secondary track, only Soller slits 2.5° opening were positioned. Samples were measured in parafocusing geometry on a zero‐background sample holder in the 2 theta range from 2 to 70°, with a step size of 0.0204° and exposure time of 0.2 s. The motorized air‐scatter knife was exploited to obtain a better signal‐to‐noise ratio at lower angles. Structural Rietveld refinements of LAG products were carried out by Topas Academic v.7 on the collected data.^[^ ^64^ ^]^

##### Hirshfeld Surfaces and Energy Framework Calculations

CrystalExplorer 21.5 was exploited to compute Hirshfeld surfaces, fingerprint plots, energy frameworks, and lattice energies for each solved structure.^[^ ^65^ ^]^ Hirshfeld surfaces were generated with a high‐resolution setting, consistently with some previous works on co‐crystals.^[^ ^66^ ^,^ ^67^ ^]^ Hirshfeld surface data are collected in ESI file (Table S2 for detailed contact list and figures S18‐S19). The wavefunctions for individual molecules and pairwise interactions for energy framework calculations were obtained using Tonto,^[^ ^68^ ^]^ exploiting the CE‐B3LYP DFT level of theory with the DGDZVP basis set.^[^ ^69^ ^]^ This combination, is identified as the optimal condition for investigating potential halogen bonding phenomena while mitigating the impact of Basis Set Superposition Error (BSSE), as outlined in the work of Siiskonen and Priimagi.^[^ ^70^ ^]^ The tube size scale for energy framework visualizations (Figure S20 in ESI) was set to 80, and the energy cutoff value was established at 0 kJ mol^−1^. Interaction energies between each molecule and its neighbors were computed, from which the sum of lattice energies for each unique molecule was derived as half the product of the number of symmetry‐equivalent molecules in the cluster and the total energy as described byThomas et al.^[^ ^57^ ^]^

##### DSC and TG

Thermal analysis—TG and DSC—were performed using simultaneous TG‐DSC analyzer Netzsch STA 449 F3 Jupiter. The samples (3–4 mg) were placed in a ceramic (Al_2_O_3_) sample holder and then heated with the heating rate of 10 °C min^−1^ from 20 to 400 °C in N_2_ atmosphere.^[^ ^71^ ^]^

##### ATR–FTIR Measurements

The ATR‐FTIR spectra of the studied compounds were collected using a Perkin Elmer Spectrum 3TM instrument with an ATR module. The spectra were recorded at room temperature in the spectral range from 4500 to 450 cm^−1^ at a resolution of 4 cm^−1^.

## Supporting Information

The Supporting Information includes additional figures and tables: complete sample list, crystal data and structure refinement for compounds **1*α***‐**3*α***, hydrogen bonds geometry for compounds **1*α***‐**3*α***, C—H···π interactions geometry for compounds **1*α***‐**3*α***, crystals of compound **1*α***‐**3*α*** (Figures S1‐S3), elemental analysis and MALDI‐TOF mass spectra for compound **1*α***‐**3*α*** and **1*β***‐**3*β*** (Figures S4‐S9), the molecular structure of compounds **1*α***‐**3*α***, crystal packing of compound **1*α***‐**3*α***, different types of packing motif of carbamazepine, Rietveld refinement for compounds **1*β***‐**3*β***, ATR‐FTIR characterization and background on Hirshfeld surface analysis.

## Conflict of Interest

The authors declare no conflict of interest.

## Supporting information

Supplementary Material

## Data Availability

The data that support the findings of this study are available from the corresponding author upon reasonable request.
