# Supplementary material for: Impact of Halogen Substituent Nature and Position on the Structural and Energetic Properties of Carbamazepine Cocrystals with Meta‐Halobenzoic Acids: A Two‐Pathway Synthesis Study
Source: Chempluschem. 2025 Sep 18;90(11):e202500474. doi: 10.1002/cplu.202500474 (PMC12605704; doi:10.1002/cplu.202500474)
Supplement: Supplementary file 1 — Supplementary Material [file CPLU-90-e202500474-s001.pdf]

Supporting information

Impact of Halogen Substituent Nature on the Structural and Energetic Properties of Carbamazepine Cocrystals with Meta-Halobenzoic Acids: A Two-Pathway Synthesis Study

Artur Mirocki[b], Mattia Lopresti[a]*

[b] Faculty of Chemistry of the University of Gdansk, ul. Wita Stwosza 63, 80-308 Gdansk, Poland
[a] Università del Piemonte Orientale, Dipartimento di Scienze e Innovazione Tecnologica, Viale T. Michel 11, 15121 Alessandria, Italy
e-mail corresponding author: artur.mirocki@ug.edu.pl, mattia.lopresti@uniupo.it

Table S1. Complete sample list.

Crystallization from solution (α)			Crystallization from LAG (β)		
Carbamazepine (g)	*Coformer*	*Weight of coformer (g)*	*Carbamazepine (g)*	*Coformer*	*Weight of coformer (g)*
0.040	2-fluorobenzoic acid	0.023	0.040	2-fluorobenzoic acid	0.023
0.040	2-chlorobenzoic acid	0.026	0.040	2-chlorobenzoic acid	0.026
0.040	2-bromobenzoic acid	0.034	0.040	2-bromobenzoic acid	0.034
0.040	2-iodobenzoic acid	0.042	0.040	2-iodobenzoic acid	0.042
0.040	3-fluorobenzoic acid	0.023	0.040	3-fluorobenzoic acid	0.023
0.040	3-chlorobenzoic acid	0.026	0.040	3-chlorobenzoic acid	0.026
0.040	3-bromobenzoic acid	0.034	0.040	3-bromobenzoic acid	0.034
0.040	3-iodobenzoic acid	0.042	0.040	3-iodobenzoic acid	0.042
0.040	4-fluorobenzoic acid	0.023	0.040	4-fluorobenzoic acid	0.023
0.040	4-chlorobenzoic acid	0.026	0.040	4-chlorobenzoic acid	0.026
0.040	4-bromobenzoic acid	0.034	0.040	4-bromobenzoic acid	0.034
0.040	4-iodobenzoic acid	0.042	0.040	4-iodobenzoic acid	0.042

Table S2. Crystal data and structure refinement for compounds **1α** - **3α**.[78]

**Compound**	**1α**	**2α**	**3α**
Chemical formula	$C_{22}H_{17}ClN_2O_3$	$C_{22}H_{17}BrN_2O_3$	$C_{22}H_{17}IN_2O_3$
Formula weight/g·mol^{-1}	392.82	437.28	484.27
Crystal system	monoclinic	monoclinic	monoclinic
Space group	$P2_1/c$	$P2_1/c$	$P2_1/c$
a/Å	15.5838(18)	15.6432(12)	15.7429(13)
b/Å	5.1299(5)	5.1396(3)	5.1377(3)
c/Å	24.594(3)	24.6877(16)	25.1161(18)
α/°	90	90	90
β/°	104.377(12)	105.170(7)	105.801(8)
γ/°	90	90	90
V/Å^3	1904.6(4)	1915.7(2)	1954.7(3)
Z	4	4	4
T/K	295(2)	295(2)	295(2)
λ_{Mo}/Å	1.54184	1.54184	1.54184
ρ_{calc}/g·cm^{-3}	1.370	1.516	1.646
F(000)	816	888	960
μ/mm^{-1}	1.992	3.142	13.086
θ range/°	3.711-61.994	3.710-67.448	3.658-67.350
Completeness *θ*/%	100.0	99.2	99.1
Reflections collected	16852	15259	16284
Reflections	2992	3413	3476
unique	[R_{int}=0.3944]	[R_{int}=0.0929]	[R_{int}=0.1059]
Data/restraints/parameters	2992/0/263	3413/0/263	3476/0/263
Goodness of fit on F^2	1.067	1.032	1.045
Final R_1 value (I>2σ(I))	0.1453	0.0534	0.0563
Final wR_2 value (I>2σ(I))	0.3774	0.1200	0.1322
Final R_1 value (all data)	0.2525	0.0945	0.0959
Final wR_2 value (all data)	0.4830	0.1525	0.1676
CCDC number	2429571	2429572	2429573

Table S3. Hydrogen bonds geometry for compounds 1-3.

Compound	D–H···A	d(D–H) [Å]	d(H···A) [Å]	d(D···A) [Å]	∠D–H···A (°)
	N(18)–H(18A)···O(27)	0.78(16)	2.20(15)	2.909(14)	151(11)
	O(26)–H(26)···O(17)	0.82(15)	1.97(17)	2.590(12)	132(15)
	C(8)–H(8)···O(27)^{i}	0.93	2.57	3.272(16)	132
	C(4)–H(4)···O(17)^{ii}	0.93	2.659	3.523	155.18
**1**	C(6)–H(6)···N(18)^{ii}	0.93	2.749	3.614	155.20
	C(23)–H(23)···O(17)^{iii}	0.93	2.674	3.534	154.01
	C(24)–H(24)···O(26)^{iii}	0.93	2.686	3.550	154.79
	C(2)–H(2)···Cl(28)^{iv}	0.93	3.016	3.715	133.14
	Symmetry code: (I) -x, -y, 1-z; (ii) x, -1+y, z; (iii) 1-x, 1-y,1-z; (iv) x, 1/2-y, 1/2+z.				
	N(18)–H(18A)···O(27)	0.82(6)	2.17(6)	2.939(6)	157(5)
	O(26)–H(26)···O(17)	0.88(7)	1.72(7)	2.591(5)	167(7)
	C(8)–H(8)···O(27)^{i}	0.93	2.60	3.298(7)	132
	C(4)–H(4)···O(17)^{ii}	0.93	2.669	3.537	155.45
**2**	C(6)–H(6)···N(18)^{ii}	0.93	2.749	3.628	157.98
	C(23)–H(23)···O(17)^{iii}	0.93	2.669	3.528	153.98
	C(24)–H(24)···O(26)^{iii}	0.93	2.710	3.555	151.47
	C(2)–H(2)···Br(28)^{iv}	0.93	3.087	3.781	132.84
	Symmetry code: (i) -x, -y, 1-z; (ii) x, -1+y, z; (iii) 1-x, 1-y,1-z; (iv) x, 1/2-y, 1/2+z.				
	N(18)–H(18A)···O(27)	0.87(10)	2.18(9)	2.935(10)	146(8)
	O(26)–H(26)···O(17)	1.04(10)	1.57(12)	2.573(8)	160(10)
	C(8)–H(8)···O(27)^{i}	0.93	2.653	3.358	133.19
	C(4)–H(4)···O(17)^{ii}	0.93	2.671	3.530	153.92
**3**	C(6)–H(6)···N(18)^{ii}	0.93	2.742	3.608	155.32
	C(23)–H(23)···O(17)^{iii}	0.93	2.676	3.536	154.03
	C(24)–H(24)···O(26)^{iii}	0.93	2.684	3.526	151
	C(2)–H(2)···I(28)^{iv}	0.93	3.250	3.953	134.02
	Symmetry code: (i) -x, -y, 1-z; (ii) x, -1+y, z; (iii) 1-x, 1-y,1-z; (iv) x, 1/2-y, 1/2+z.				

Table S4. C–H···π interactions geometry for compounds 1-3.

Compound	C–H···Cg[a]	d(H···Cg) [Å]	d(C···Cg) [Å]	∠C–H···Cg (°)
	C(10)–H(10)···Cg1^{ii}	3.073	3.714	127.49
**1**	C(11)–H(11)···Cg2^{ii}	3.092	3.740	128.18
		Symmetry code: (ii) x, -1+y, z.		
	C(10)–H(10)···Cg1^{ii}	3.112	3.747	127.11
**2**	C(11)–H(11)···Cg2^{ii}	3.072	3.711	127.43
		Symmetry code: (ii) x, -1+y, z.		
	C(10)–H(10)···Cg1^{ii}	3.123	3.746	125.92
**3**	C(11)–H(11)···Cg2^{ii}	3.081	3.727	128.08
		Symmetry code: (ii) x, -1+y, z.		

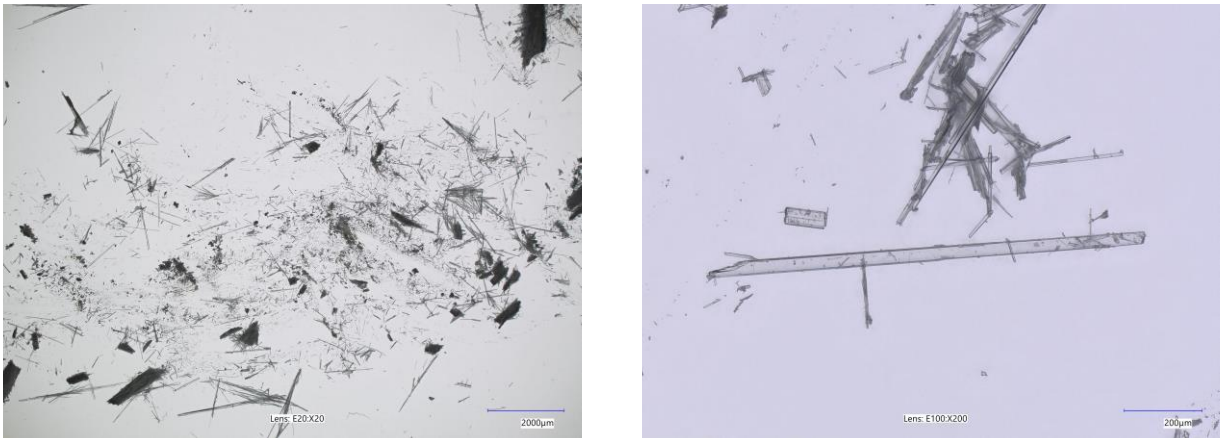

Figure S1. Crystals of compound 1α.

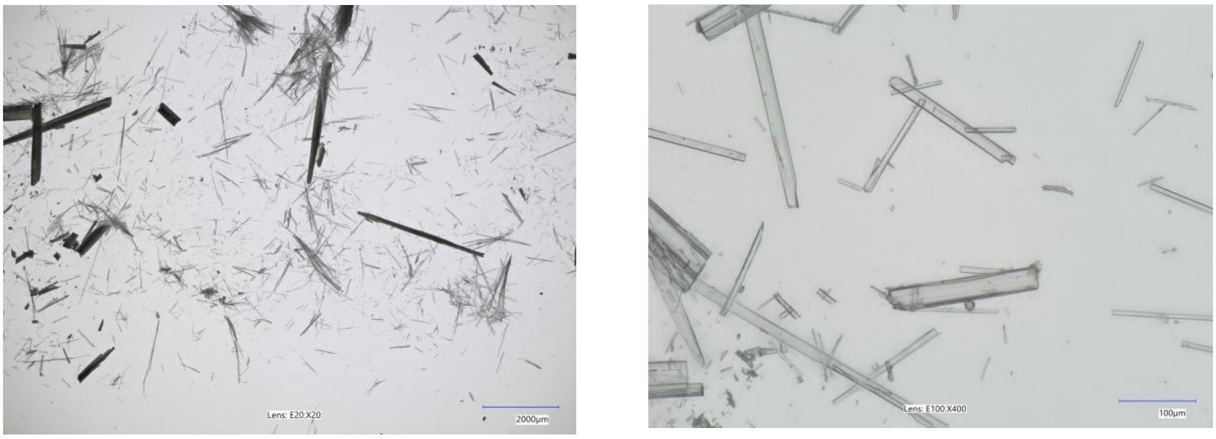

Figure S2. Crystals of compound 2α.

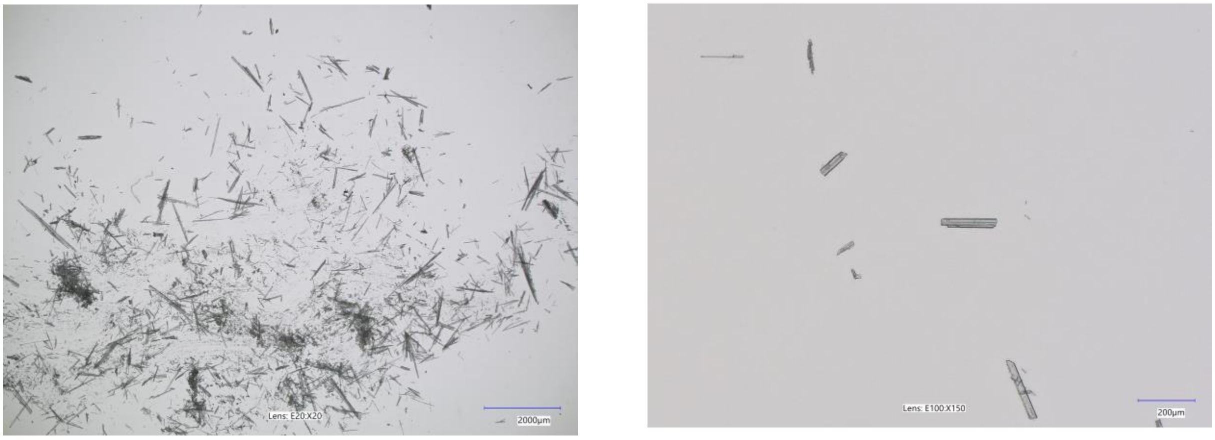

Figure S3. Crystals of compound 3α.

*Elemental analysis of compound **1α-3α**:*

a) compound **1α**: subtotal mass calculated for C: 67.26%, found: 67.80%, calculated for H: 4,36%, found: 4.48%, calculated for N: 7.13%, found: 7.82%
b)
c) compound **2α**: subtotal mass calculated for C: 60.43%, found: 59.21%, calculated for H: 3.92%, found: 3.79%, calculated for N: 6.41%, found: 5.95%

d) compound **3α**: subtotal mass calculated for C: 54.56%, found: 51.67%, calculated for H: 3.54%, found: 3.32%, calculated for N: 5.78%, found: 4.89%

MALDI-TOF mass spectra for compound **1α-3α**: calculated for $[C_{15}H_{13}N_2O]^+$: 237.103; found for compound **1α**: 237.029; found for compound **2α**: 237.050; found for compound **3α**: 237.055 (Figure S4-S6).

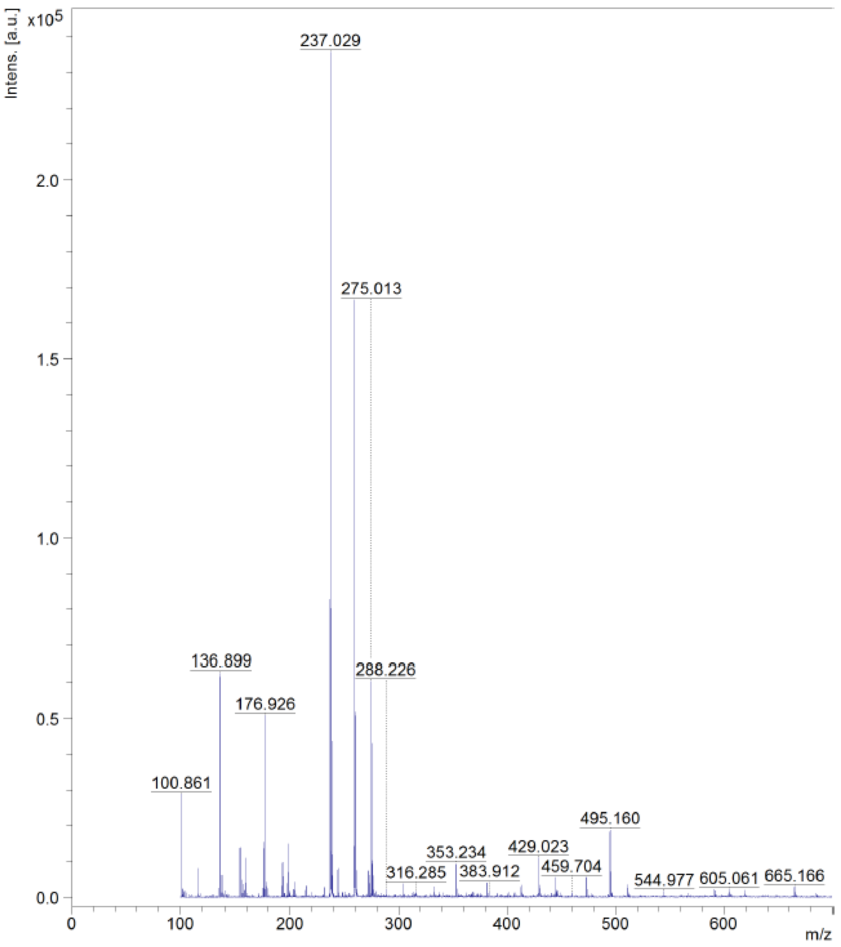

Figure S4. MALDI-TOF mass spectra for compound 1α (calculated for $[C_{15}H_{13}N_2O]^+$: 237.103; found: 237.029). Positive-ion mode MALDI-TOF mass spectra were obtained using a Bruker Biflex III spectrometer.

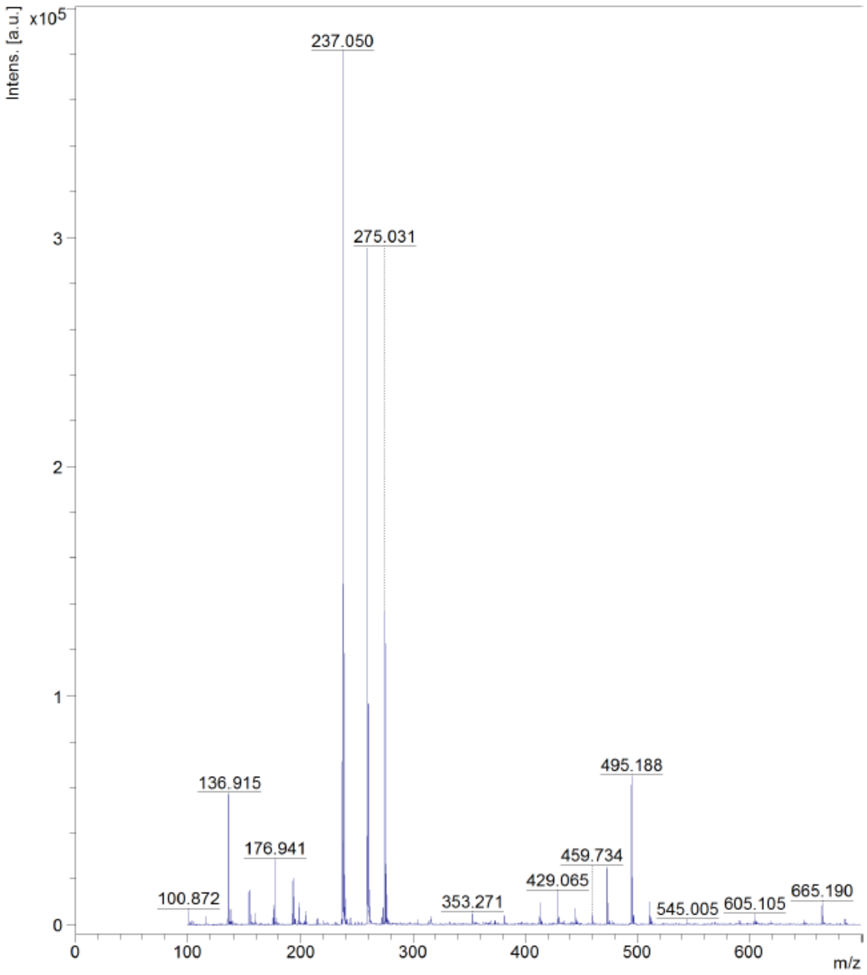

Figure S5. MALDI-TOF mass spectra for compound 2α (calculated for $[C_{15}H_{13}N_2O]^+$: 237.103; found: 237.050). Positive-ion mode MALDI-TOF mass spectra were obtained using a Bruker Biflex III spectrometer.

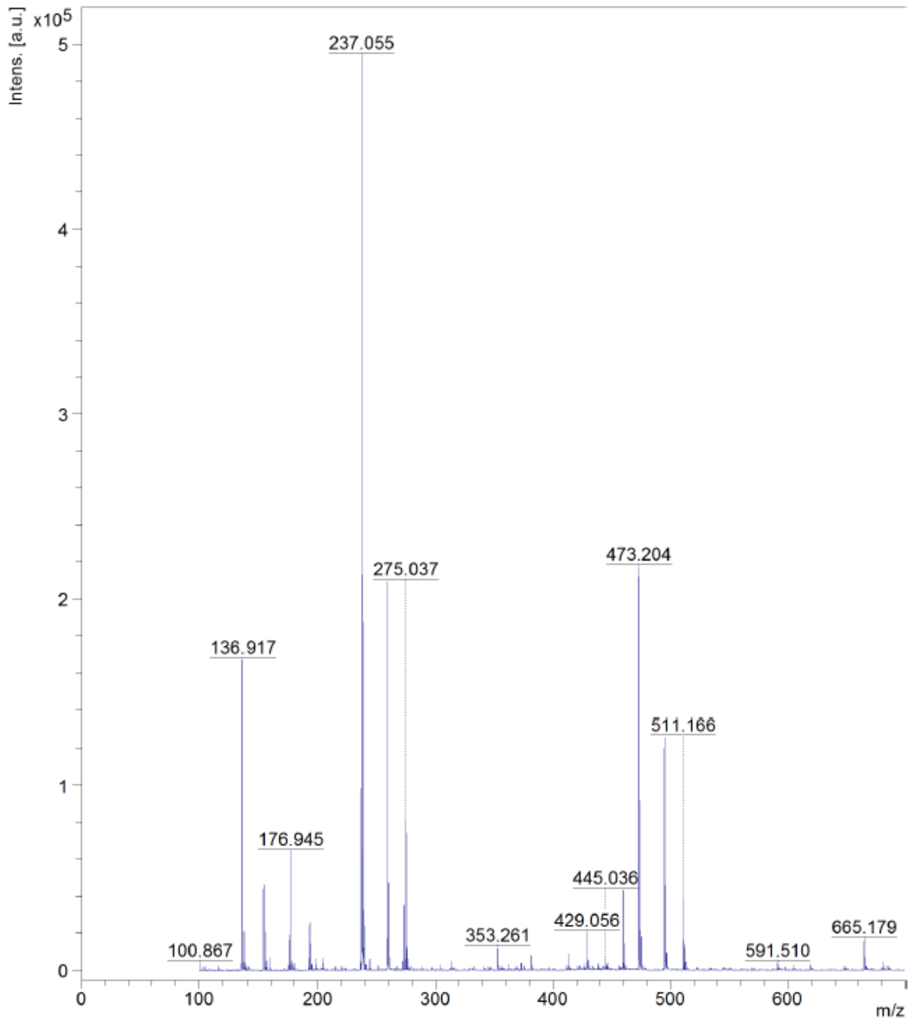

Figure S6. MALDI-TOF mass spectra for compound 3α (calculated for $[C_{15}H_{13}N_2O]^+$: 237.103; found: 237.055). Positive-ion mode MALDI-TOF mass spectra were obtained using a Bruker Biflex III spectrometer.

*Elemental analysis of compound **1β-3β***

a) compound **1β**: subtotal mass calculated for C: 67.26%, found: 65.91%, calculated for H: 4,36%, found: 4.63%, calculated for N: 7.13%, found: 6.21%

b) compound **2β**: subtotal mass calculated for C: 60.43%, found: 59.96%, calculated for H: 3.92%, found: 3.97%, calculated for N: 6.41%, found: 6.21%

c) compound **3β**: subtotal mass calculated for C: 54.56%, found: 54.43%, calculated for H: 3.54%, found: 3.67%, calculated for N: 5.78%, found: 5.70%

MALDI-TOF mass spectra for compound **1β-3β**: calculated for $[C_{15}H_{13}N_2O]^+$: 237.103; found for compound **1β**: 237.056; found for compound **2β**: 237.053; found for compound **3β**: 237.055 (Figure S7-S9).

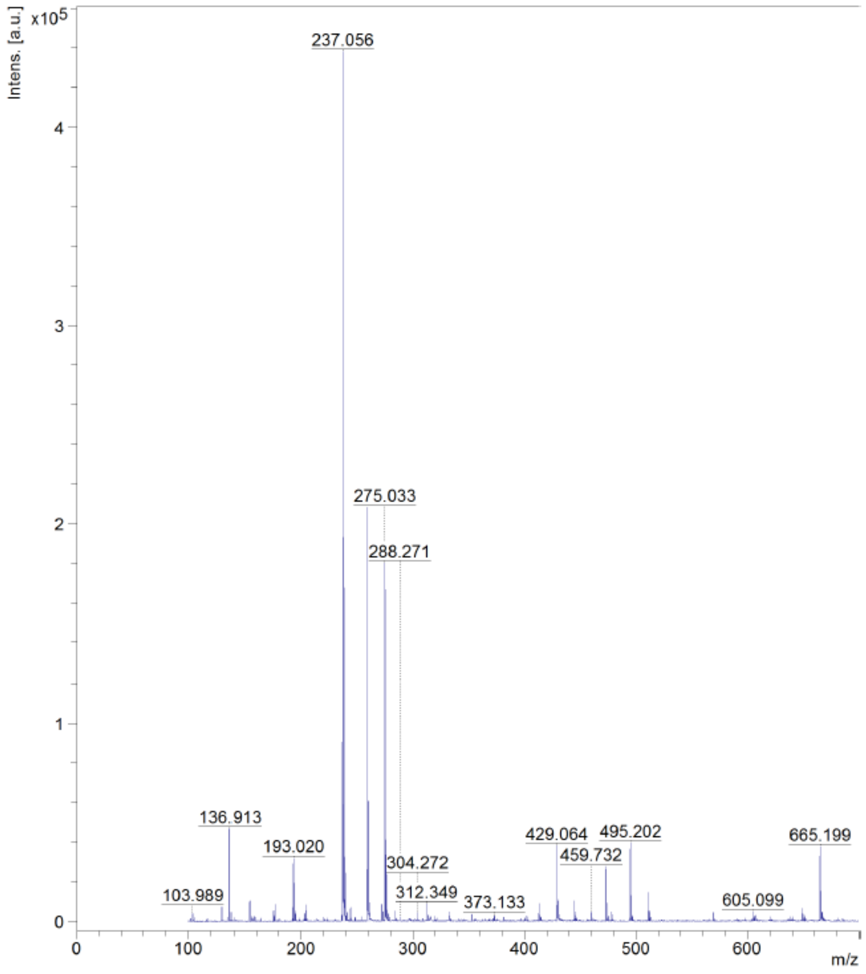

Figure S7. MALDI-TOF mass spectra for compound 1β (calculated for $[C_{15}H_{13}N_2O]^+$: 237.103; found: 237.056). Positive-ion mode MALDI-TOF mass spectra were obtained using a Bruker Biflex III spectrometer.

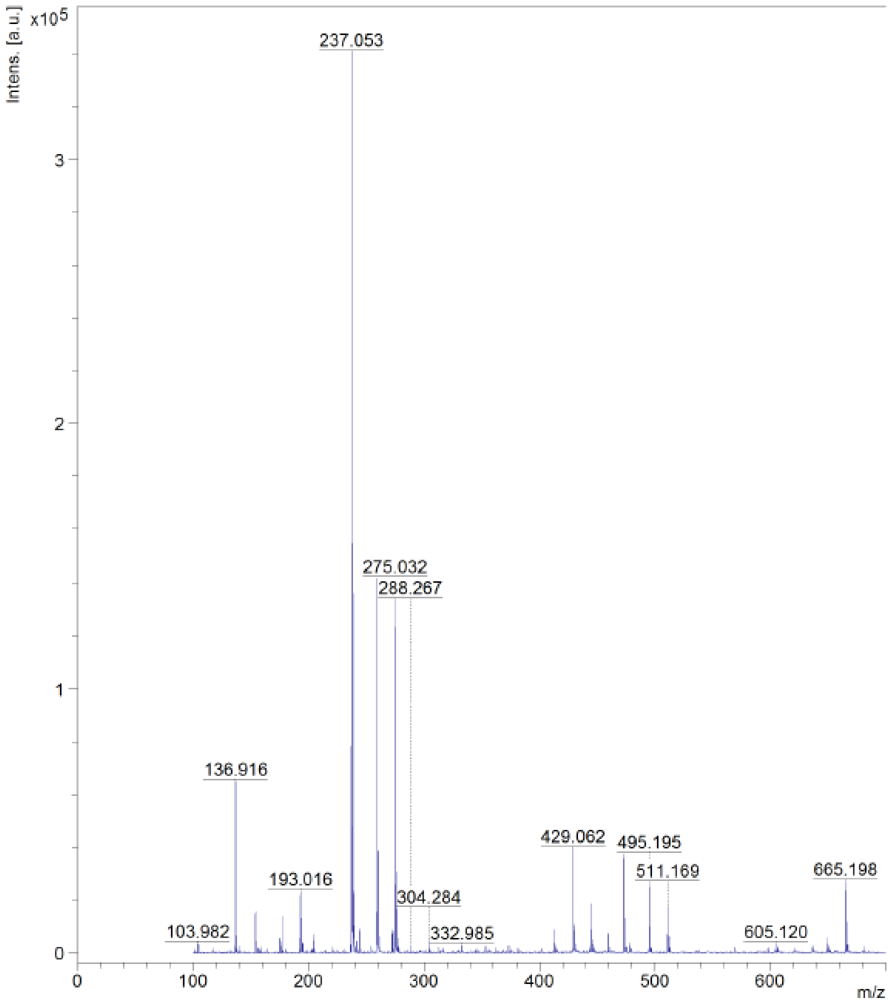

Figure S8. MALDI-TOF mass spectra for compound 2β (calculated for $[C_{15}H_{13}N_2O]^+$: 237.103; found: 237.053). Positive-ion mode MALDI-TOF mass spectra were obtained using a Bruker Biflex III spectrometer.

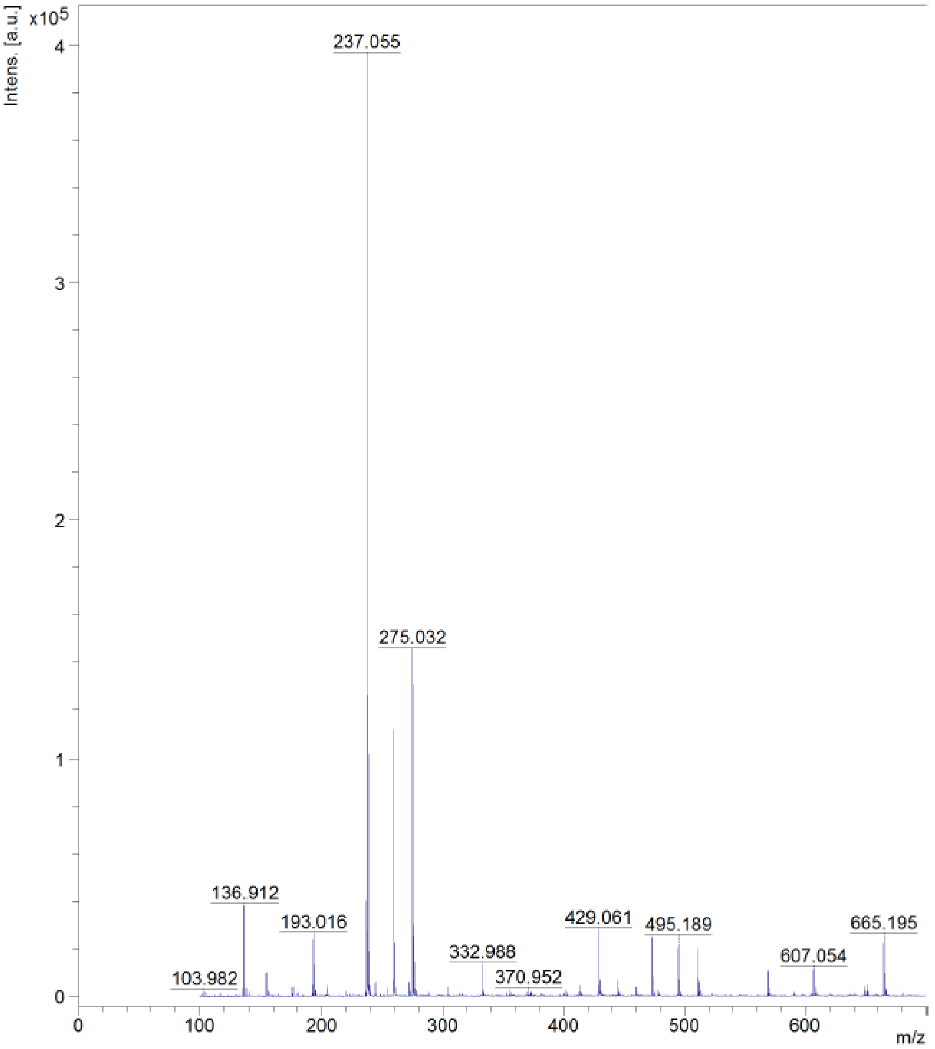

Figure S9. MALDI-TOF mass spectra for compound 3β (calculated for $[C_{15}H_{13}N_2O]^+$: 237.103; found: 237.055). Positive-ion mode MALDI-TOF mass spectra were obtained using a Bruker Biflex III spectrometer.

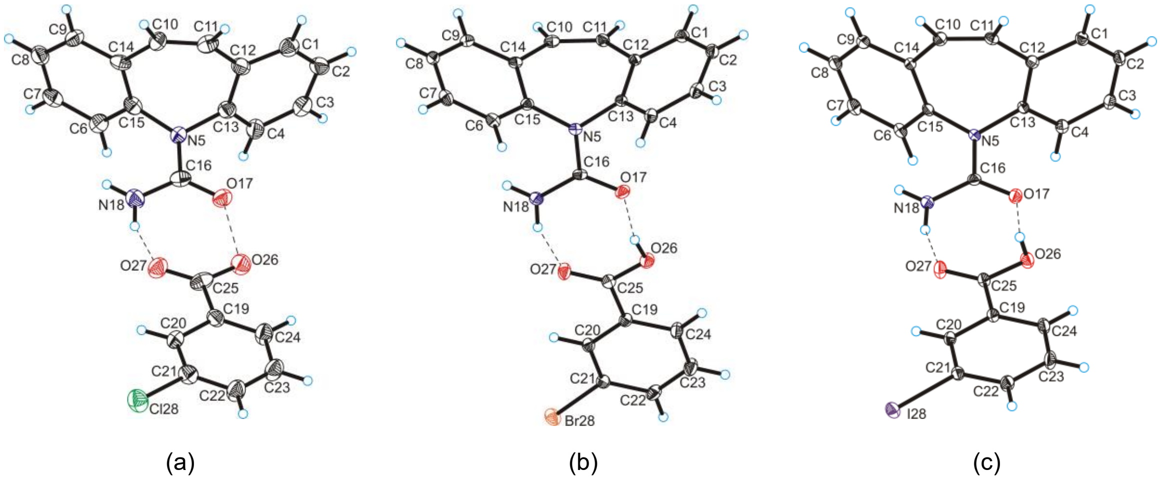

Figure S10. The molecular structure of compounds 1α–3α in a-c, respectively, showing the atom-labelling scheme. The hydrogen bonds are represented by dashed lines.

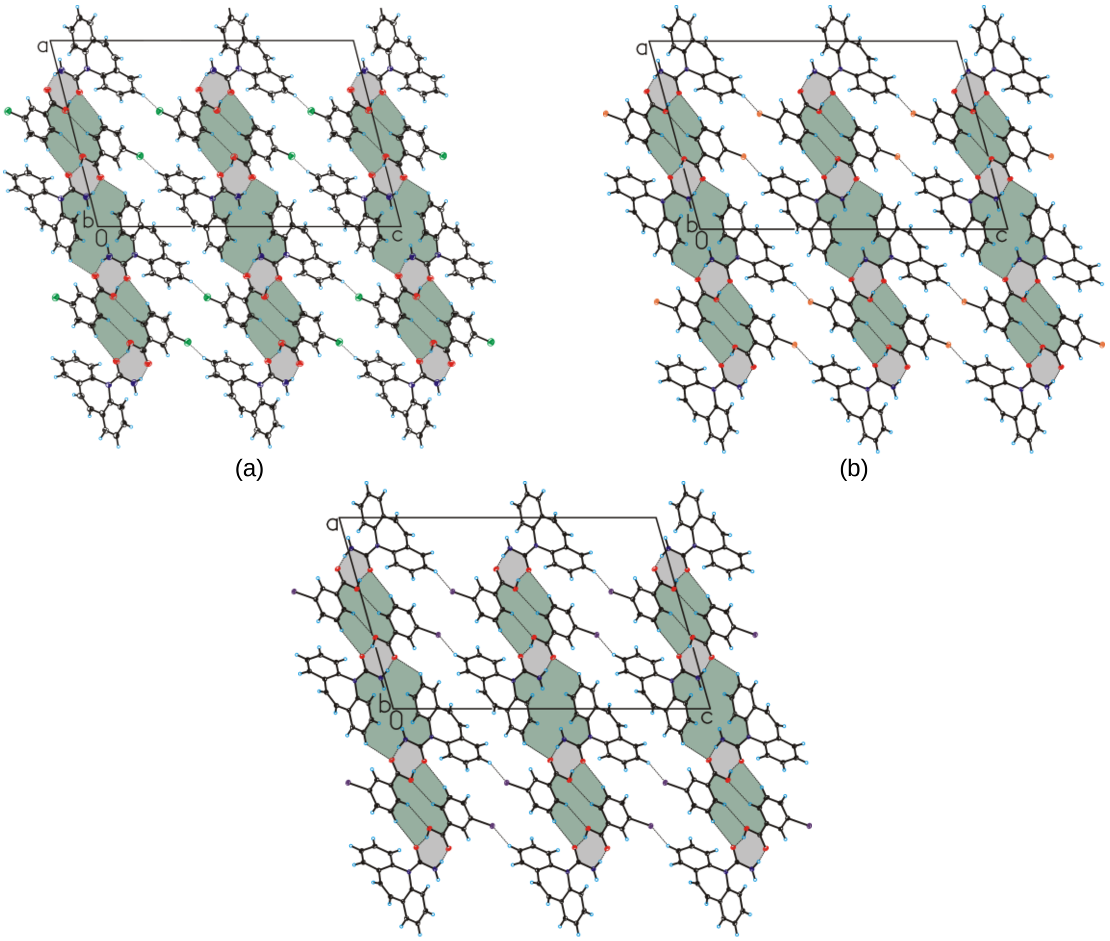

(a) (b)

(c)

Figure S11. Crystal packing of compound 1α-3α in a-c, respectively. The hydrogen bonds are represented by dashed lines.

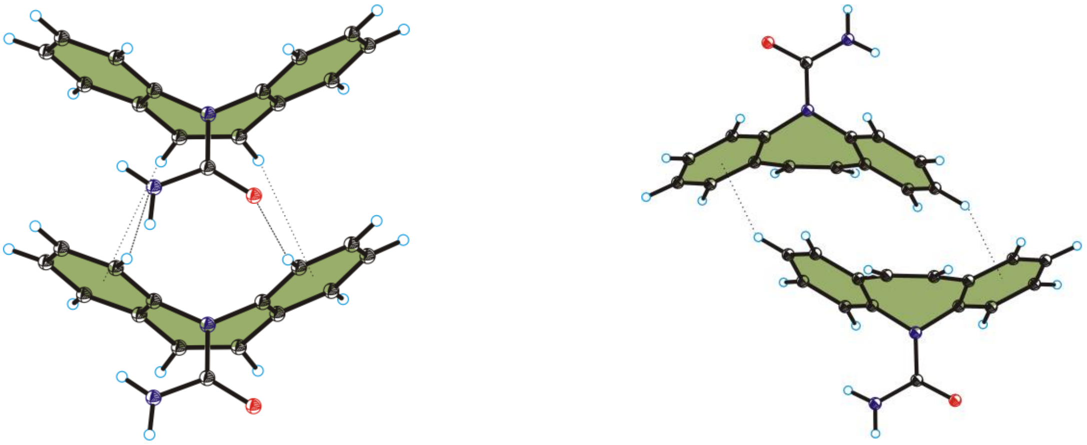

(a) (b)

Figure S12. Childs et al. showed that carbamazepine can adopt different types of packing motif such as the translation stack interaction, inversion cup interaction and coformer pairing interaction.[62] (a) The translation stack motif involving carbamazepine molecules (REFCODE: XOXHEY)[62], (b) inversion cup motif involving carbamazepine molecules (REFCODE: MOXVIF)[62].

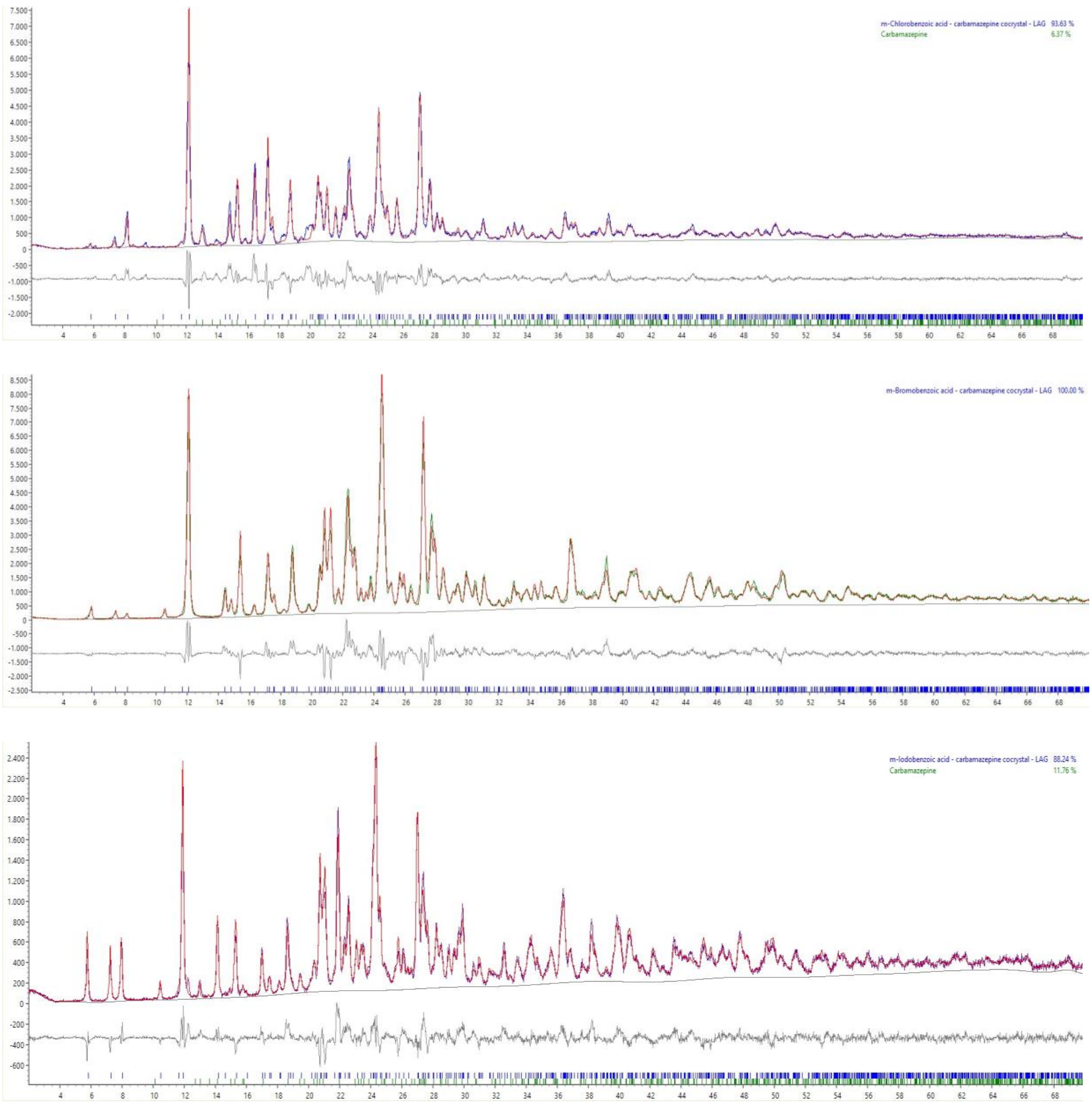

Figure S13. Rietveld refinement for compounds **1β**, **2β** and **3β**, based on structures obtained by SCXRD data from compounds **1α**, **2α** and **3α**.

ATR-FTIR characterization

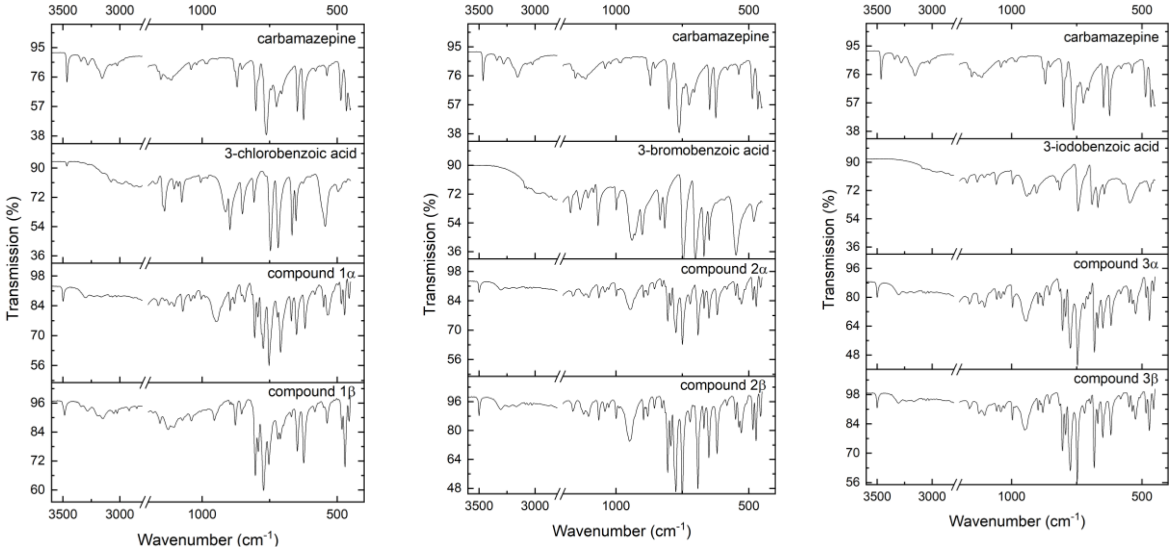

(a) (b) (c)

Figure S14. The ATR-FTIR spectra in the range of 3500–400 cm^{-1} of (a) carbamazepine and 3-chlorobenzoic acid; (b) carbamazepine and 3-bromobenzoic acid; (c) carbamazepine and 3-iodobenzoic acid.

In Figure S14, all collected ATR-FTIR spectra are reported. The bands from 3500 to 3165–3164 cm^{-1} region of the ATR-FTIR spectrum of crystal containing 3-halobenzoic acid and carbamazepine can be assigned to the free and hydrogen-bonded asymmetric and symmetric stretching vibrations of N–H bonds. The broad absorption in the range of 3300–2500 cm^{-1} is attributed to the O–H stretching vibration of the carboxylic group, which is typically broadened due to hydrogen bonding. The O–H band strongly obscures the stretching vibrations of =C–H in the 3100–3000 cm^{-1} region. The C=O stretching vibration observed at 1690 cm^{-1} for compound **1(α** and **β)** and **2(α** and **β)**, and 1687 cm^{-1} for compound **3(α** and **β)** is blue-shifted by approximately 10 cm^{-1} compared to pure 3-chloro-, 3-bromo- and 3-iodobenzoic acid and carbamazepine. Moreover, a new medium intensity band at 1632 cm^{-1} or 1634 cm^{-1} appearing only for the cocrystals can be assigned to the amide C=O stretching vibrations or N–H amide group deformation. The appearance of this band is attributed to a reorganization of the hydrogen bonding network relative to the pure substrates. Additionally, the IR peaks observed in the 1600–1400 cm^{-1} range correspond to C=C stretching vibrations of the aromatic ring, which causes the vibrations in spectra to overlap. Peaks in the 1161–1056 cm^{-1} range correspond to C–N and C–O stretching vibrations, while those in the 1000–600 cm^{-1} range correspond to =C–H out-of-plane bending vibrations. However, the broad peak at 949 cm^{-1} for compound **1(α** and **β)** and for compound **2(α** and **β)** and **3(α** and **β)** can also be attributed to the out-of-plane bending of the O-H group in the carboxylic acid, engaged in hydrogen

bonding. The C–Cl, the C–Br and C–I stretching vibration are observed in the 850–550 cm^{-1}, 690–515 cm^{-1} and 600–500 cm^{-1} region, respectively for compound **1(α** and **β)**, **2(α** and **β)** and **3(α** and **β)**. Comparing the ATR spectra of single crystals and powders of the title compounds, it is visible that the results obtained are identical, which also confirms that both pathways for obtaining compounds **1**, **2** and **3** lead to the same products.

Structural superposition of SC-XRD solved cocrystals and XRPD sovled cocrystals.

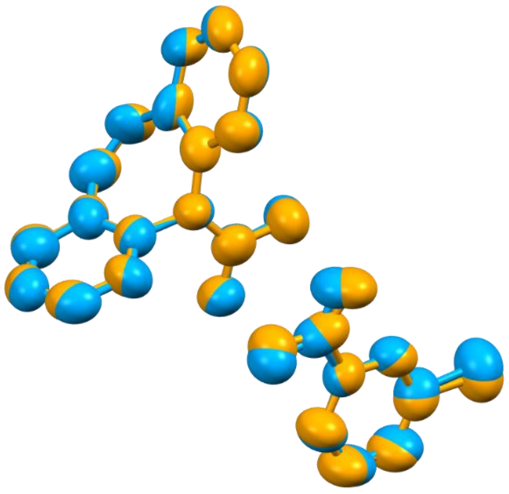

Figure S15. Superposition between the asymmetric units of the SC-XRD solved cocrystal of the chloro- derivative (blue) and the XRPD solved one (orange).

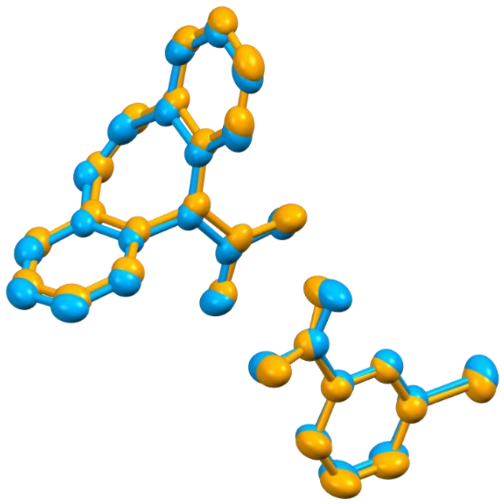

Figure S16. Superposition between the asymmetric units of the SC-XRD solved cocrystal of the bromo- derivative (blue) and the XRPD solved one (orange).

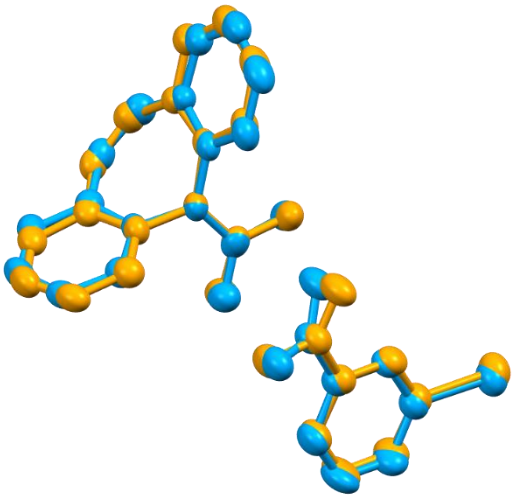

Figure S17. Superposition between the asymmetric units of the SC-XRD solved cocrystal of the iodo- derivative (blue) and the XRPD solved one (orange).

Background on Hirshfeld surface analysis

The Hirshfeld surface is reported calculated using the d_{norm} value, which is the normalized contact distance. All d_{norm} were calculated as:[66]

$$d_{norm} = \frac{(d_i - d_i^{vwd})}{d_i^{vdw}} + \frac{(d_e - d_e^{vdw})}{d_e^{vdw}}$$

where d_i^{vdw} and d_e^{vdw} represent the Van der Waals radii of the atoms inside and outside the surface respectively, and di and de are the separations between the closest atoms inside and outside the surface. The 3D d_{norm} surfaces are presented as a colour gradient: red patches denote inter-contacts participating in intermolecular interactions, while blue zones represent regions where neighbouring atoms are too distant to interact. Originating from the Hirshfeld surface, 2D-fingerprint plots are generated. Fingerprint plots visually condense the occurrence frequency of various d_i and d_e combinations throughout a molecule's surface. Consequently, they not only pinpoint the presence of intermolecular interactions but also illustrate the proportional distribution of each interaction type across the surface area.

Table 2. Percentage of the interactions among all the possible pairs of atoms in the structures. Carbamazepine values are constant for each structure; therefore, it's reported only once.

Molecule	C···C	C···H	C···N	C···O	C···X	H···C	H···H	H···N	H···O	H···X	N···C	N···H	N···N	N···O	N···X
m-chlorobenzoic acid	3.6%	8.8%	0.0%	3.8%	2.6%	6.1%	30.0%	0.0%	8.3%	2.0%	-	-	-	-	-
m-bromobenzoic acid	3.7%	8.5%	0.0%	3.6%	2.8%	5.8%	29.4%	0.0%	7.9%	2.1%	-	-	-	-	-
m-iodobenzoic acid	3.6%	8.3%	0.0%	3.3%	2.8%	5.9%	28.1%	0.0%	7.5%	2.2%	-	-	-	-	-
carbamazepine	1.3%	19.5%	0.0%	0.4%	0.0%	14.5%	44.3%	0.7%	6.3%	4.7%	0.0%	0.7%	0.0%	0.5%	0.0%

Molecule	O···C	O···H	O···N	O···O	O···X	X···C	X···H	X···N	X···O	X···X	C ···all	H ···all	N ···all	O ···all	X ···all
m-chlorobenzoic acid	2.4%	11.7%	0.8%	0.4%	0.2%	2.6%	15.7%	0.0%	0.3%	0.7%	18.8%	46.4%	-	15.5%	19.3%
m-bromobenzoic acid	2.4%	11.5%	0.7%	0.4%	0.1%	3.2%	16.2%	0.0%	0.3%	1.4%	18.6%	45.2%	-	15.1%	21.1%
m-iodobenzoic acid	2.2%	11.3%	0.7%	0.3%	0.1%	3.6%	17.7%	0.0%	0.3%	2.1%	18.0%	43.7%	-	14.6%	23.7%
carbamazepine	0.6%	6.3%	0.0%	0.2%	0.0%	-	-	-	-	-	21.2%	70.5%	1.2%	7.1%	-

Carbamazepine

In Figure S18, the Hirshfeld surface and element-filtered fingerprint plots of carbamazepine are presented. Notably, the most prominent short contacts identified are N–H···O and O···H–O, indicative of interactions occurring at the on-the-plane interface between the amidic group of carbamazepine and the carboxylic group of the acid. This confirms that the strongest interaction inside the structure is given by the formation of the heterosynthon with the benzoic acid counterpart. Additionally, the presence of a homosynthon involving two carbamazepine molecules is noteworthy. This synthon, characterized by N–H···O hydrogen bonds between adjacent carbamazepine molecules, is frequently observed in various carbamazepine cocrystals and polymorphs, contributing to their structural stability, as reported in the previous sections. The competition between the carbamazepine···carbamazepine homosynthon and the carbamazepine···acid heterosynthon plays a crucial role in dictating the final crystalline arrangement, with the latter being favoured due to the strong carboxyl-amide interactions. Interestingly, due to the specific packing arrangement of the molecules, a carbamazepine/m-halobenzoic acid system exhibits equivalent configurations situated directly above and below the referenced arrangement. Consequently, other notable short interactions are observed, such as H6···N' and H4···O', further contributing to the understanding of the molecular interactions and particular stability within this system.

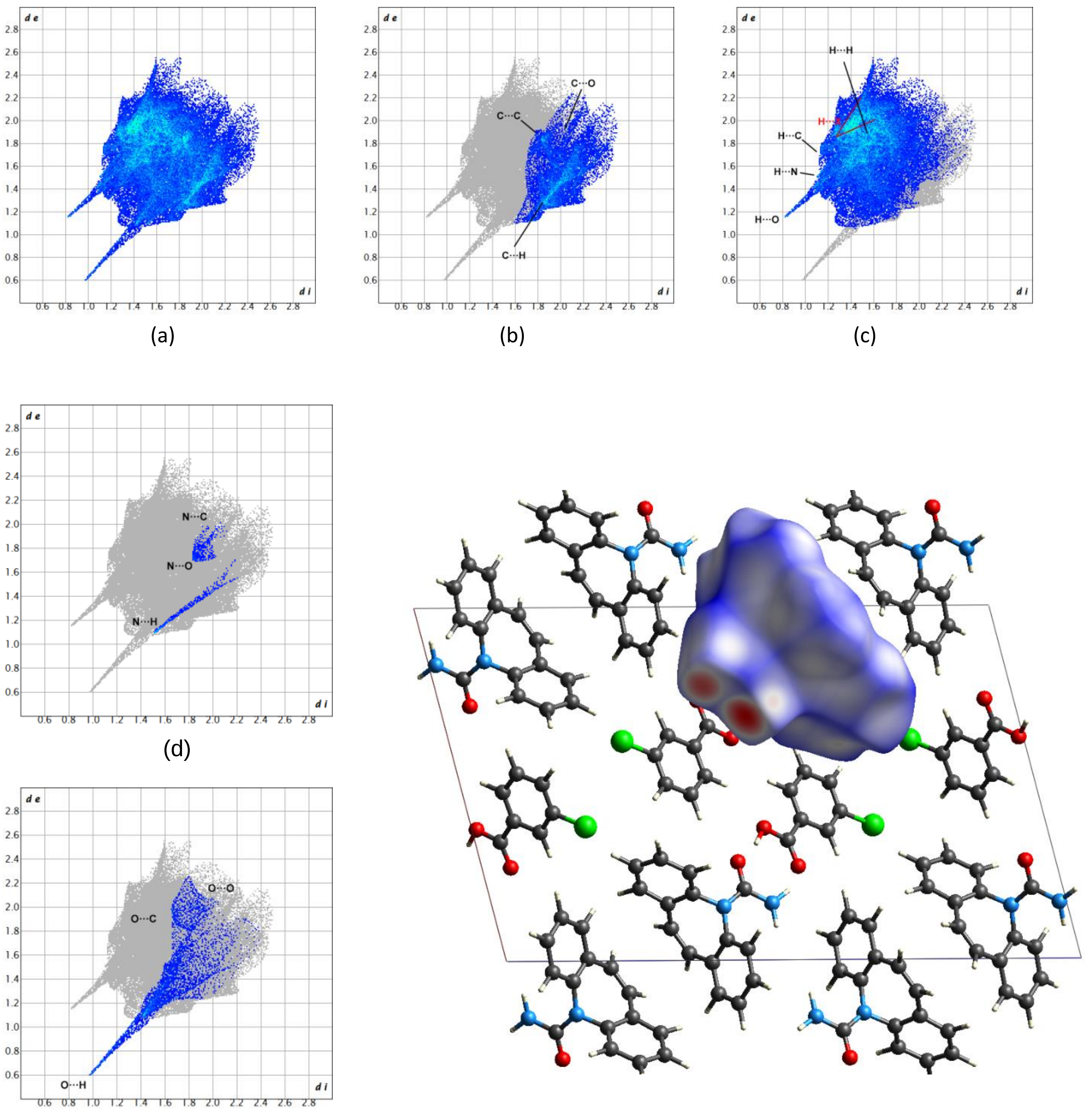

(a) (b) (c)

(d)

(e)

(f)

Figure S18. Hirshfeld surface and fingerprint plots of carbamazepine: (a) complete fingerprint plots; (b) filtered for C contacts; (c) filtered for H contacts; (d) filtered for N contacts; (e) filtered for O contacts; (f) Hirshfeld surface highlighted for d-norm projected on carbamazepine and viewed along the b-axis.

<u>Meta-halobenzoic acids</u>

The Hirshfeld surfaces highlighted for d-norm of the three acids appear visually like each other, but with color tones transitioning from deep blue to white proportionally with the r_X as defined in the previous sections. This can be easily observed in Figure S19. In the bottom row of Figure S19, the relative shape index surfaces are also reported for a better visualization of the hue changes in the surface of the three halobenzoic acids.

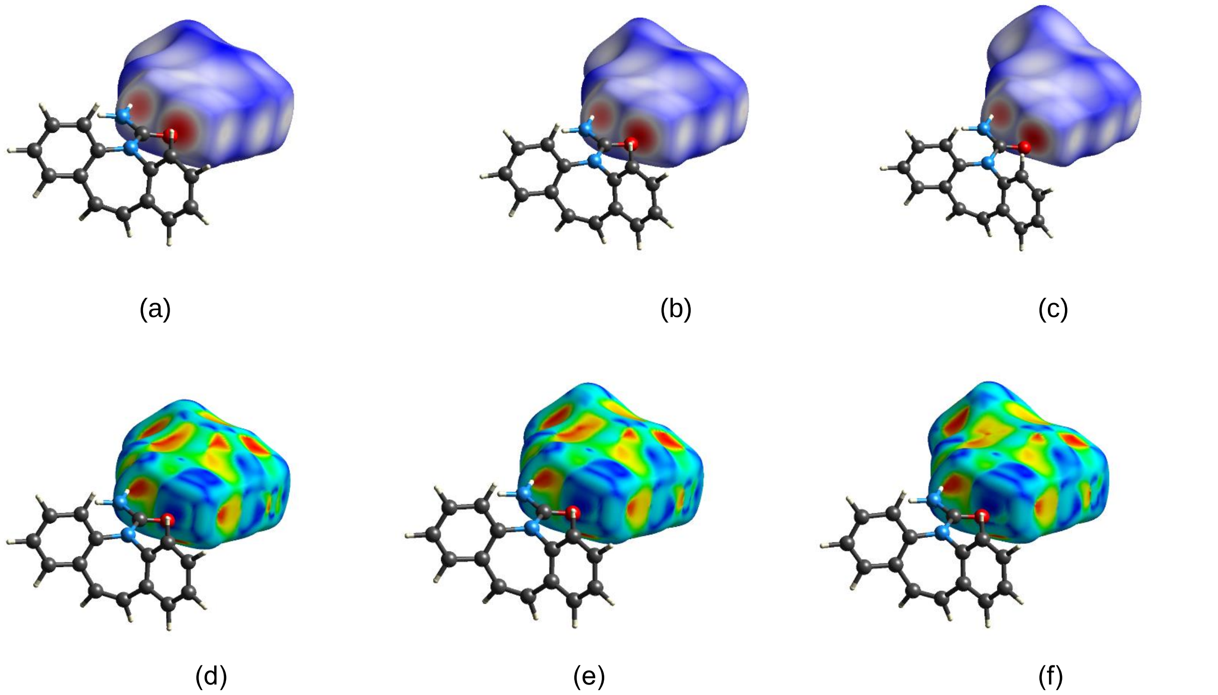

Figure S19. Hirshfeld surfaces projected on the three m-halobenzoic acids: (a, d) Hirshfeld surface of m-chlorobenzoic acid, (b, e) Hirshfeld surface of m-bromobenzoic acid, (c, f) Hirshfeld surface of m-iodobenzoic acid. Top row: surface highlighted for d-norm; bottom row: surface highlighted for shape index.

Energy frameworks, visualized as cylinders representing interaction energies, aid in analyzing supramolecular architecture. These frameworks reveal anisotropy in molecular packing, crucial for understanding mechanical behavior like bending and shearing in crystals. Overall, energy frameworks provide a powerful tool to comprehend the complex interplay of forces influencing molecular crystal structures in various directions. In Figure S20, the energy frameworks calculated for **1** are depicted. The energy frameworks of the other two isostructural co-crystals are not shown as they appear to be perfectly identical in appearance. It can be observed that the contributions due to Coulomb interactions are positioned in the interface plane between the carboxylic and amide groups, confirming a strong electrostatic interaction. Of lesser magnitude, but still significant, are the Coulomb interactions between carbamazepine molecules along the translation axis. Along the same direction, cylinders representing the highest lattice dispersion energies are observed. The two combinations of the two parameters, reported in Figure S20, illustrate how the total energy is predominantly oriented in the h = 1, k = 0, l = -1 plane and that there are areas where interactions are weaker.

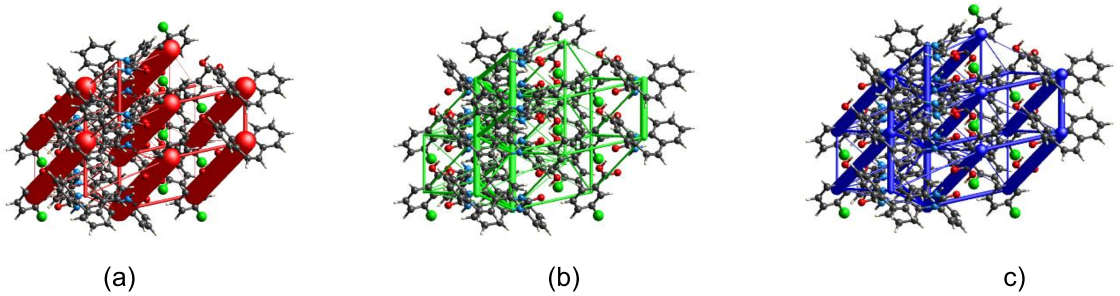

Figure S20. Energy frameworks for the carbamazepine/m-chlorobenzoic acid (**1**) solved structure. a) Coulomb energy; b) Dispersion energy; c) Total energy. Only the for m-chlorobenzoic acid cocrystal is reported, as the bromine and iodine derivatives have the same energy frameworks.
